# Linkage disequilibrium network analysis (LDna) gives a global view of chromosomal inversions, local adaptation and geographic structure

**DOI:** 10.1111/1755-0998.12369

**Published:** 2015-01-21

**Authors:** Petri Kemppainen, Christopher G Knight, Devojit K Sarma, Thaung Hlaing, Anil Prakash, Yan Naung Maung Maung, Pradya Somboon, Jagadish Mahanta, Catherine Walton

**Affiliations:** * Computational and Evolutionary Biology, Faculty of Life Sciences, University of ManchesterManchester, UK; † Institute of Vertebrate Biology, Academy of Sciences of the Czech RepublicBrno, Czech Republic; ‡ Regional Medical Research Centre, NE (ICMR)Dibrugarh, 786 001, India; § Department of Medical Research (Lower Myanmar), Medical Entomology Research Division5 Ziwaka Road, Dagon P.O., Yangon, 11191, Myanmar; ¶ Department of Parasitology, Faculty of Medicine, Chiang Mai UniversityChiang Mai, 50200, Thailand

**Keywords:** *Anopheles dirus*, A*nopheles gambiae*, chromosomal rearrangement, graph theory, landscape genomics, r package

## Abstract

Recent advances in sequencing allow population-genomic data to be generated for virtually any species. However, approaches to analyse such data lag behind the ability to generate it, particularly in nonmodel species. Linkage disequilibrium (LD, the nonrandom association of alleles from different loci) is a highly sensitive indicator of many evolutionary phenomena including chromosomal inversions, local adaptation and geographical structure. Here, we present linkage disequilibrium network analysis (LDna), which accesses information on LD shared between multiple loci genomewide. In LD networks, vertices represent loci, and connections between vertices represent the LD between them. We analysed such networks in two test cases: a new restriction-site-associated DNA sequence (RAD-seq) data set for *Anopheles baimaii*, a Southeast Asian malaria vector; and a well-characterized single nucleotide polymorphism (SNP) data set from 21 three-spined stickleback individuals. In each case, we readily identified five distinct LD network clusters (single-outlier clusters, SOCs), each comprising many loci connected by high LD. In *A. baimaii*, further population-genetic analyses supported the inference that each SOC corresponds to a large inversion, consistent with previous cytological studies. For sticklebacks, we inferred that each SOC was associated with a distinct evolutionary phenomenon: two chromosomal inversions, local adaptation, population-demographic history and geographic structure. LDna is thus a useful exploratory tool, able to give a global overview of LD associated with diverse evolutionary phenomena and identify loci potentially involved. LDna does not require a linkage map or reference genome, so it is applicable to any population-genomic data set, making it especially valuable for nonmodel species.

## Introduction

Recent developments in next-generation sequencing (Davey *et al*. 2011, 2013; Seeb *et al*. 2011) have opened up a new era of population genomics in nonmodel species, broadening the range of evolutionary and ecological questions that can be addressed (Andrew *et al*. 2013; Narum *et al*. 2013). A major aim in this field is to distinguish locus-specific effects (such as selection) from genomewide effects (such as population structure and demographic history). This is often achieved by identifying outlier loci in empirical distributions of population-genetic statistics such as polymorphism and divergence (Gaggiotti *et al*. 2009; Fisher *et al*. 2011). Considering loci separately like this ignores potentially valuable information about alleles from multiple loci that may be nonrandomly associated with each other, that is be in linkage disequilibrium (LD; Hill & Robertson 1968; Barton 2011).

LD exists when combinations of alleles across loci deviate from well-mixed (statistical equilibrium) expectations (Barton *et al*. 2007). Thus, any evolutionary phenomenon that perturbs the system away from this equilibrium, such as population structure or selection, will leave a signature of LD in the genome. Once LD exists, any mechanism that modulates its decay (i.e. affects the rate of recombination), such as chromosomal rearrangements (Rieseberg 2001) or recombination cold/hot spots (Maniatis 2002) will also leave its mark in patterns of LD. Most notably, inversions strongly restrict recombination in heterokaryotypes, in particular around the inversion break points (Noor & Bennett 2009). LD therefore has the potential to be informative about many important evolutionary phenomena that affect genomes (Ardlie *et al*. 2002; Slatkin 2008).

Many current methods to analyse genomewide multilocus LD require the genomic position of the loci to be known (International HapMap Consortium 2005; Voight *et al*. 2006; Falush *et al*. 2007; Kim *et al*. 2008; Kumasaka *et al*. 2010; Lawson *et al*. 2012; Koch *et al*. 2013; Ralph & Coop 2013) and are therefore limited to species with well-annotated reference genomes. This is unfortunate as the ability to gain information about LD associated with important evolutionary phenomena does not crucially depend on knowing where the loci come from in the genome. The focus on using genomic location means that while measures of LD may in principle be applied to loci across the genome, they are frequently only applied within chromosomes, or to specific subsets of chromosomes (e.g. the MHC locus). This loses information about LD among more widely scattered loci. To address these issues, we develop here a network-analytical approach to identifying groups of loci with high intragroup LD. It does not require knowledge of the physical position of loci in the genome and can be used for all loci from a population-genomic data set in a single analysis. Appropriate population-genetic analyses of the sets of loci identified by our approach may then reveal their involvement in evolutionary phenomena, enabling a novel global view of processes shaping the genome.

Here, we will use networks to refer to the combinations of vertices and edges which form the heart of mathematical graph theory. Network analyses have successfully been used to study a diverse range of complex biological processes (Mason & Verwoerd 2007; Foote *et al*. 2009; Knight & Pinney 2009; Marbach *et al*. 2010). A central theme in network analyses is to identify sets of vertices (clusters) that have more and/or stronger connections between their members than to the remainder of the network (Newman & Girvan 2004; Leskovec *et al*. 2009). In our network-analytical approach to LD, the vertices in a network represent loci and the edges between them represent LD. In this way, we will use all pairwise LD values among loci to gain an overall picture of LD within a given population-genomic data set.

Any evolutionary phenomena that result in elevated LD among multiple loci are expected to cause distinct clusters in LD networks. Some examples, such as inversions and selective sweeps, only affect localized genomic regions within single chromosomes. Others involve loci more widely spread in the genome, potentially spanning several chromosomes. These include epistatic (nonadditive) fitness interactions among loci and population admixture. Admixture LD can be natural, for example the recent rejoining of allopatrically diverged populations; or it can be artificial, for example where the study sample comprises individuals from two or more divergent populations. In both cases, drift or selection, acting independently in the ancestral or sampled populations respectively, will result in sets of loci sharing high LD, potentially scattered across the genome. When such different evolutionary phenomena responsible for LD co-occur and are sufficiently different from each other, that is do not affect the same individuals or loci in the same way, we expect each to generate a distinct cluster in an LD network.

To identify clusters of loci that share high LD within an LD network, we have developed linkage disequilibrium network analysis (LDna). We evaluate the LDna approach by applying it to two study systems exhibiting well-characterized evolutionary phenomena associated with elevated LD among multiple loci: inversions, local adaptation and geographic structure. The first of these is *Anopheles baimaii,* a mosquito which is a major malaria vector in Southeast Asia (Sinka *et al*. 2011; Sarma *et al*. 2012). *Anopheles baimaii* has a widespread distribution extending from northeast India, through Myanmar and into Thailand (Obsomer *et al*. 2012). Polytene chromosome studies have identified five large inversions, each on a different chromosomal arm (2L, 2R, 3L, 3R and the X-chromosome; Baimai *et al*. 1988a,b; Poopittayasataporn & Baimai 1995). These inversions are polymorphic within populations, occurring at varying frequencies across the distribution of this species (Baimai *et al*. 1988a,b; Poopittayasataporn & Baimai 1995). We thus predict that in a population-genomic data set from this species, LDna will identify distinct clusters of loci, each cluster corresponding to an inversion.

The second system is the well-studied three-spined stickleback (*Gasterosteus aculeatus*; Colosimo *et al*. 2005; Jones *et al*. 2012). In this species, we expect, in addition to three known inversions, local adaptation to marine and freshwater habitats and geographical structuring between the Atlantic and Pacific populations to be associated with LD signals among multiple loci. Population-genomic data from this species will enable us to evaluate the extent to which LDna is able to detect distinct clusters associated with the simultaneous presence of different evolutionary phenomena.

## Materials and methods

### Linkage disequilibrium network analysis (LDna) outline

**Fig. 1:**
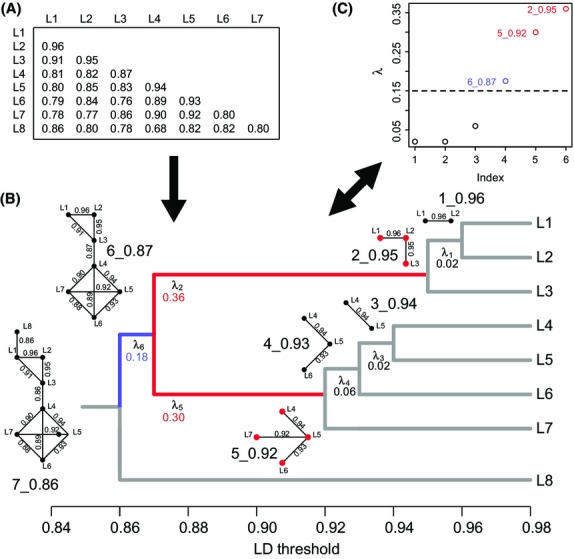
Outline of linkage disequilibrium network analysis (LDna). (A) Starting from a pairwise matrix of LD values between loci, LDna partitions all loci into clusters comprising vertices (loci) connected by edges that represent LD values above given thresholds. (B) The order in which clusters merge with decreasing threshold can be visualized as a tree where only one connection between clusters is required for clusters to be considered as merged. For each cluster in the tree, the change in median LD of all pairwise connections between loci in a cluster at merger is measured by *λ* (see Materials and methods). (C) All lambda values plotted in order of increasing value (Index). Clusters with exceptionally high values of *λ* relative to the median across all the values in a tree (above the, user-controlled, dashed line) are considered as outliers. In (B) and (C), red colour highlights clusters that do not have any other outlier clusters nested within them (single-outlier clusters, SOCs), and blue highlights the outlier cluster that contains multiple SOCs (compound outlier cluster, COC).

An outline of LDna is given in Fig.1. We start with a matrix of pairwise LD values (Fig.1A). LD was measured as the squared pairwise correlation coefficient between loci, *r*^2^ (Hill & Robertson 1968), calculated using the ‘LD’ function in the r package ‘genetics’ (Warnes *et al*. 2013). These LD values were treated as weights for edges that connect loci (vertices) in networks which were constructed using the r package ‘igraph’ (Csardi & Nepusz 2006). We generate a series of networks, each using the subset of pairwise LD values above a particular threshold. As LD threshold decreases, vertices become increasingly connected in clusters that grow and eventually merge to form a single fully connected network. This successive merging of clusters can be effectively visualized as a tree (Fig.1B), where branches represent clusters and the joining of branches represents clusters and/or individual loci merging (i.e. become connected by at least one edge) at a particular LD threshold.

The change in LD when two clusters merge is measured by *λ* (Fig.1B,C). We calculated *λ* for every cluster in the tree, defined as: 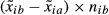, where 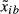 is the median of all intracluster *r*^2^ values for cluster *i* before merger; after merger 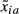 is the median of intracluster *r*^2^ values for those pairwise LD values involving at least one locus from the premerger cluster *i*; and *n*_*ib*_ is the number of loci in cluster *i* before merger. High values of *λ* indicate the merger of large clusters or strongly associated clusters, that is where intracluster pairwise LD values are high relative to intercluster LD values (Fig.1B). Any *λ* value exceeding the median by a multiple *φ* of the median absolute deviation and containing at least |*E*|_min_ edges is designated an outlier cluster (Fig.1C). The two parameters, *φ* and |*E*|_min_, allow the user to pick out both ‘diffuse’ and ‘compact’ clusters as outliers. A diffuse cluster can be made up of many moderately associated and moderately connected vertices, while a compact cluster has a few vertices with strong associations and/or high connectivity. The purpose of these parameters is to enable the identification of clusters representing sets of loci that bear distinct evolutionary genetic signals in the data. Approaches to parameter value choice are explored in Results and in Appendix S1 and S2 (Supporting Information; these are also included as tutorials for the r package ‘LDna’, see Data accessibility).

From the outlier values identified, we wish to determine the subsets that correspond to discrete evolutionary phenomena. In practice, we observe that some outlier clusters are nested within others. We designate any ‘tip’ cluster with no other cluster nested within it as a single-outlier cluster (SOC, coloured red in Fig.1). Any other outlier we designate as a compound outlier cluster (COC, coloured blue in Fig.1). The set of SOCs identified in this way represents mutually exclusive clusters, each containing unique loci that share high LD. We hypothesize that each SOC corresponds to a distinct evolutionary phenomenon acting in the population. If this is the case, COCs may contain information about the relationships among evolutionary phenomena. However, exploring the interpretation of COCs is beyond the scope of this study where we shall focus on testing the biological interpretation of SOCs.

### Population-genetic interpretation of LDna analysis on simulated data

**Fig. 2:**
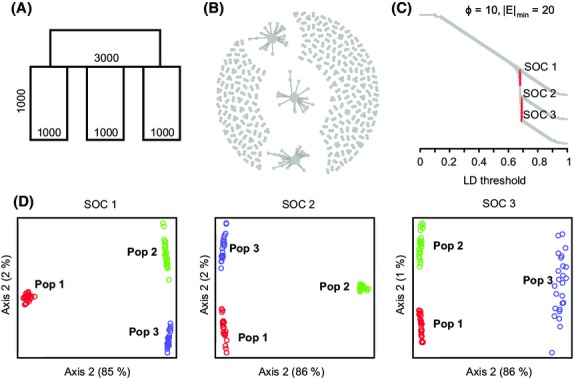
LDna on data simulated for a subdivided population. (A) Outline of modelled scenario where an ancestral population splits into three populations followed by 1000 generations of independent evolution. (B) Resulting LDna network showing clusters formed above an LD threshold of 0.8 (C) Tree showing LD clusters across LD thresholds (comparable to Fig.1B). LDna identified three SOCs, highlighted in red, at the parameter values shown. (D) PCAs for each of the three SOCs identified in (C). The amount of variation explained is indicated on each axis.

To illustrate how LDna may be applied to more realistic data, we created a data set simulated under a scenario of population structure using fastsimcoal2 (Excoffier & Foll 2011; Excoffier *et al*. 2013; see Appendix S3, Supporting Information, for detailed methods). This involved an ancestral population that split into three populations, each with effective population size of 1000 diploid individuals, 1000 generations ago (Fig.2A). These populations evolved through mutation, recombination and drift only, without selection or migration (see Appendix S3, Supporting Information, for details). LDna was applied to 25 diploid individuals from each final population.

Populations were pooled prior to calculating LD, thereby creating sample admixture LD. As expected for three equivalent populations, LDna identifies three SOCs at similar LD thresholds (Fig.2B,C). Analysis of these SOCs by PCA reveals that each SOC represents the genetic distinction of each population from the other two due to the unique trajectory of mutation and drift in each population (Fig.2D). This pattern, in which the number of clusters corresponds to the number of comparisons among populations, can be seen for other numbers of simulated populations too (Appendix S3-Fig2, Supporting Information). When we incorporate migration among populations into these simulations, the resulting recombination erases the LD clusters progressively with increasing migration rate (Appendix S3-Fig3, Supporting Information).

**Fig. 3:**
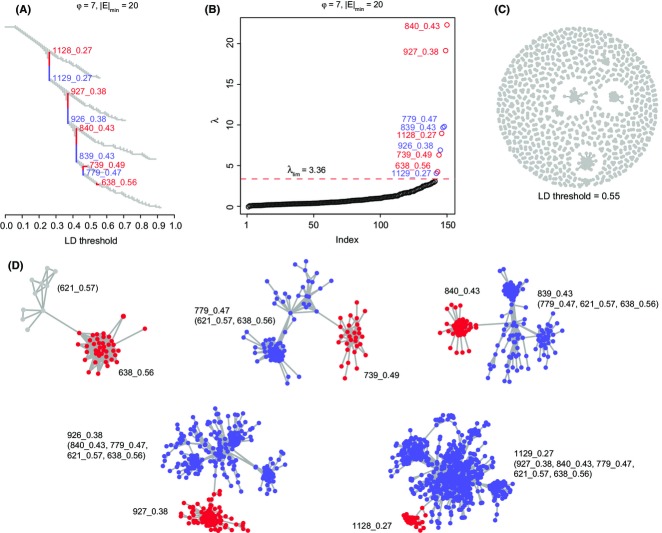
LDna of *Anopheles baimaii* RAD sequence data set. (A) A clustering tree (cf. Figs1B and 2C) of all pairwise *r*^2^ values from 3828 SNPs derived from a landscape genomics RAD sequence data set from *A. baimaii*. Branches corresponding to SOCs and COCs are indicated in red and blue, respectively, throughout the figure. (B) All *λ* values in increasing order with values above *λ*_lim_ corresponding to outlier clusters. Parameter values for *φ* and |*E*|_min_ are shown above plots (A) and (B). See Fig.6 and Appendix S1 and S2, Supporting Information, for details of parameter value selection. (C) A snapshot of a full network at an LD threshold value just above that at which any of the five SOCs merge. (D) Each SOC is shown at an LD threshold where it is joined by a single link to other loci, in decreasing order of threshold from left to right, top to bottom. For each of these mergers, we have indicated, in brackets after the COC name, which SOCs are nested within each COC. COCs are shown here but were not analysed further.

### Preparation of population-genomic data sets and genome mapping

The preparation of a restriction-site-associated DNA (RAD) population-genomic data set for *A. baimaii* and a three-spined stickleback SNP data set is described in Appendix S4 (Supporting Information). Note that when many SNPs come from the same RAD locus, they may themselves cause clustering in LDna, in particular when parameter settings for |*E*|_min_ and *φ* are set to low values (see Appendix S2 for details). However, in practice, we found that most RAD loci contained a single SNP (see Results). The consensus sequences for each relevant RAD locus were mapped against the *A. dirus* reference genome using BLAT (Kent 2002) run with the default parameters, and a *P*-value threshold of 1×10^-8^ was used to identify significant hits. Second, we mapped all our linkage map RAD loci (as above) to the scaffolds from the first step and used these to anchor the scaffolds to the linkage maps. Sequences were aligned to the *A. gambiae* genome using the blast algorithm through https://www.vector/base.org/blast with default settings except that the maximum *E*-value was set to 1×10^-3^.

As a draft genome is only available for a close relative of *A. baimaii* (*A. dirus*; estimated divergence time from *A. baimaii* ∼1 Mya; Morgan *et al*. 2010), we also produced a linkage map for *A. baimaii* (described in Appendix S4, Supporting Information). Each relevant locus was mapped against the *A. dirus* reference genome using BLAT (Kent 2002) run with the default parameters. A *P*-value threshold of 1 × 10^−8^ was used to identify significant hits, and scaffolds with positive hits were then anchored to the linkage map. Chromosomal rearrangements are very common in Diptera, but chromosome arms remain syntenic even between distantly related species (Bolshakov 2002). Therefore, we also mapped all relevant loci to the genome of *A. gambiae* (the closest well-annotated reference genome to *A. baimaii*) using blast (https://www.vector/base.org/blast) with default settings except that the maximum *E*-value was set to 1 × 10^−3^.

### Population-genetic structure

Principal component analysis (PCA) and discriminant analysis of principal components (DAPC) were implemented in the r package ‘adegenet’ (Jombart & Ahmed 2011). For PCA, first, allele frequencies were scaled and missing genotype data were replaced by the mean using function ‘scaleGen’, and the PCA was performed with function ‘dudi.pca’. For DAPC, the number of genetically distinct groups (*k*) present was first identified by running the function ‘find.clusters’, in which the function ‘kmeans’ is run sequentially with increasing number of groups and the different clustering solutions compared using the Bayesian information criterion (BIC). The optimal numbers of clusters were inferred visually by inspecting how BIC decreased as the number of groups increased following guidelines in the documentation for Adegenet. All other basic population-genetic parameters were calculated with functions from Adegenet.

## Results

### LDna reveals five clusters of high LD in *Anopheles baimaii* populations

There are five known polymorphic inversions in *Anopheles baimaii* (see Introduction). Due to the restricted recombination in heterokaryotypes, a polymorphic inversion partitions the genetic information (created by mutation, drift and/or a selective sweep) in that genomic region into two groups: the ancestral and the inverted. Consequently, each polymorphic inversion is expected to create strong admixture LD among the inversion loci. We therefore predict that any inversion for which different karyotypes (hetero- or homokaryotypes) have been sampled should give rise to a SOC in population-genomic data. To test this hypothesis, we generated and analysed a restriction-site-associated DNA (RAD) sequence data set from 224 wild-caught individuals of *A. baimaii,* sampled throughout its distribution range. Our RAD sequence data set comprised 3008 loci from 184 individuals sampled from 91 geographical sites (Fig. S1). As *r*^2^ can only be calculated between biallelic loci, we extracted all such SNPs from each RAD locus with a minor allele frequency above 10%. The data set used for subsequent LDna analyses comprised 3828 SNPs (median number of SNPs per RAD = 1, range 1–36).

Application of LDna to the above data set resulted in the identification of five SOCs (Fig.3A; Table 1). These SOCs were named 638_0.56, 739_0.49, 840_0.43, 927_0.38 and 1128_0.27, where the numbers before and after the underscore indicate a unique cluster number and the highest LD threshold at which a SOC is present, respectively. Figure3B shows that each SOC constitutes a clear outlier with respect to *λ*. Figure3C gives a snapshot of cluster formation at an LD threshold where all SOCs are visible although some are small. Figure3D gives a network visualization of the successive merging of the SOCs.

**Table 1:** Summary of single-outlier clusters (SOCs) identified by LDna of the population-genomic data sets from *Anopheles baimaii* and three-spined stickleback

Data set	SOC	*n*_loci_	|*E*|	*λ*	Median LD (MAD)*	Inferred cause
*A. baimaii*	638_0.56	40	388	4.24	0.554 (0.106)	Inversion
	739_0.49	29	68	6.30	0.334 (0.095)	Inversion
	840_0.43	67	936	22.3	0.389 (0.128)	Inversion
	927_0.38	101	925	19.1	0.227 (0.0919)	Inversion
	1128_0.27	46	364	8.96	0.211 (0.0940)	Inversion
Stickleback	494_0.82	41	278	18.8	0.797 (0.0877)	Inversion/local adaptation
	495_0.82	343	2324	18.2	0.382 (0.153)	Local adaptation
	496_0.82	25	77	13.8	0.736 (0.0920)	Inversion/local adaptation
	618_0.79	235	1263	59.8	0.416 (0.123)	Geographic structure
	673_0.76	289	526	54.0	0.255 (0.123)	Geographic structure

a Median of all intracluster pairwise LD values (*r*^2^); MAD is the median absolute deviation (unscaled).

### Hypothesis that SOCs correspond to inversions in *Anopheles baimaii*

To determine which, if any, of the five SOCs identified above correspond to inversions, we applied conventional population-genetic approaches. Lack of recombination within inversion heterokaryotypes is expected to result in genetic divergence at loci within the rearrangement, particularly those near to inversion break points. If a SOC marks an inversion, we therefore expect to be able to identify three genetically distinct groupings corresponding to the two alternative homokaryotypes and the heterokaryotype. Further, we expect the heterokaryotypic genetic groups to be genetically intermediate to the two homokaryotype groupings and to display a strong excess of heterozygous genotypes.

### Population-genetic analyses support the inversion hypothesis

**Fig. 4:**
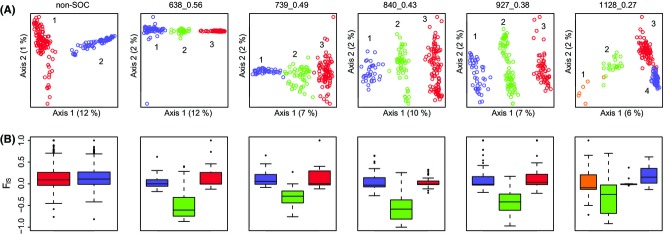
Population-genetic analyses of LD clusters from *Anopheles baimaii*. (A) For the non-SOC loci and each set of SOC loci, individuals were separated into genetically distinct groups (see main text and Fig. S2 for details) and coloured according to these groups. In (A), the separation of these groups (numbered 1–4) is visualized along the first two PCA axes, with per cent variation explained indicated on the axes. (B) The distribution of *F*_IS_ values for loci from each group indicated in (A).

Analysis of the non-SOC loci showed strong support for two genetically distinct groups (Fig. S2 and Fig.4A). This pattern serves as a null hypothesis to which population structure at the SOCs can be compared.

Four SOCs (638_0.56, 739_0.49, 840_0.43 and 927_0.38) all differed from the non-SOC loci in having strong support for three genetically distinct groups (Fig. S2 and Fig.4A). For these SOCs, DAPC found that a large proportion of the variation between these groups (>99.5%) was explained by the first discriminant function. As a result, for these SOCs, one group is intermediate between the other two. These intermediate groupings all show a strong excess of heterozygotes as indicated by highly negative values of the inbreeding coefficient, *F*_IS_ (Fig.4B). In contrast, the distributions of *F*_IS_ values for the other two groups are centred close to zero. These results are consistent with the inversion hypothesis such that groups 1 and 3 for these four SOCs represent alternative homokaryotypes and group 2 for each SOC represents heterokaryotypic individuals.

SOC 1128_0.27 showed a different pattern to the four described above. While there were still three major groups (Fig. S2), the first discriminant function explained much less of the variation among groups (77%). Four groups better partitioned the variation in *F*_IS_ and it is therefore shown in Fig.4. Similar to groups 1 and 2 of the non-SOC loci, groups 3 and 4 have nonnegative *F*_IS_ values (Fig.4B). In contrast, group 2 shows negative *F*_IS_ and is intermediate between group 1 and groups 3 and 4, consistent with group 2 being heterokaryotypic. We therefore hypothesize that SOC 1128_0.27 corresponds to a relatively rare inversion where group 1 is the low-frequency homokaryotype and groups 3 and 4 are the high-frequency homokaryotype, detected as two groups for some other reason, for example due to geographical structuring.

### Mapping locates inversions to different chromosomal arms

**Fig. 5:**
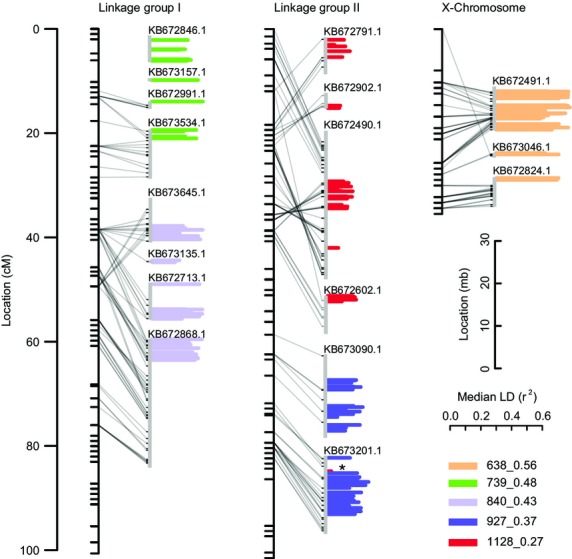
Mapping of SOC loci. For each linkage group, the linkage map (from *Anopheles baimaii*) is shown to the left and genomic scaffolds (from *A. dirus*) to which SOC loci map are shown to the right. Accession numbers are given above each scaffold. Horizontal bars indicate the positions of loci, coloured according to the figure key. Length of the bars indicates the median of all intracluster *r*^2^ values. The asterisk indicates one locus from SOC 1128_0.27 that mapped far from all other loci from this SOC. Two scaffolds for SOC 739_0.48 (top left corner) could not be anchored to the linkage maps.

The hypothesis that the five SOCs identified in *A. baimaii* correspond to five large polymorphic inversions in this species (see Introduction) further predicts that all loci from a given SOC will map together to distinct but large genomic regions. We tested this using a linkage map for *A. baimaii* (Appendix S5 and Fig.5). Loci from the above SOCs mapped to 17 different *A. dirus* scaffolds of which 15 could be anchored to the *A. baimaii* linkage map. There is broad colinearity between the linkage map and the scaffolds (Fig.5). However, there may also be rearrangements between the species, suggested by the crossing of lines between the linkage map and scaffold in Fig.5, particularly in the upper portion of linkage group II.

Loci from each of the five SOCs mapped to between two and four unique scaffolds (Fig.5). Each SOC maps to large but distinct genomic regions: two each on linkage groups I and II, respectively, and one on the X-chromosome (Fig.5). Only one locus (1 of 46 in SOC 1128_0.27) mapped away from the other loci in its SOC. For each of the five SOCs, between 96% and 100% of all BLAST hits against the *A. gambiae* genome (*n* = 7–47 per SOC) place each SOC on a different chromosome arm. SOC loci could colocate to a genomic region for several reasons, for example recombination cold spots such as telomeres or centromeres following admixture. However, given the consistency with previous cytological data (see Introduction), the observation that the SOCs map to the five large chromosome arms adds further support to the population-genetic analyses above in favour of the inversion hypothesis.

### Identification of SOCs is robust to parameter choice and data set size

**Fig. 6:**
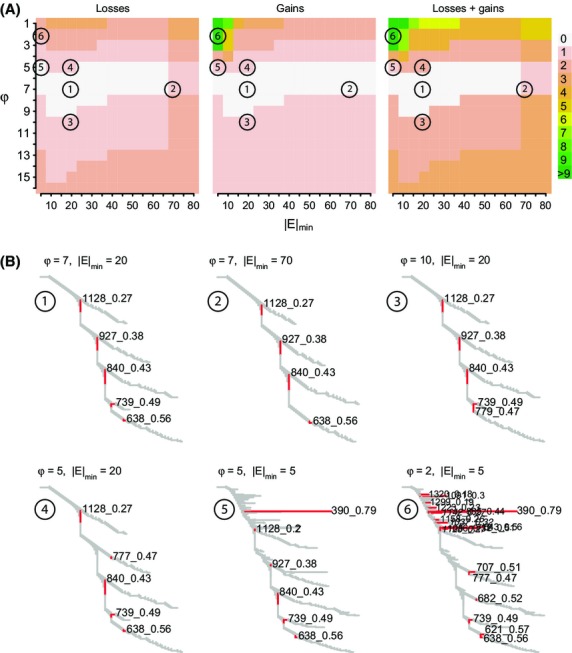
The effects of parameter choice on LDna. The two user-defined input parameters for LDna are *φ*, which controls when clusters are defined as outliers, and |*E*|_min_, the minimum number of edges required for a cluster to be considered as an outlier. (A) We used the results from the original LDna analyses (that identified five SOCs associated with inversions) as a reference point ①. With respect to this reference, we assessed how many of the SOCs were not identified (losses), and how many additional SOCs were identified (gains) by LDna. White indicates parameter space where results exactly matched the reference. In addition to the reference (Tree ①), (B) shows five examples of LDna results (Trees ②–⑥) at different combinations of *φ* and |*E*|_min_ as indicated above the trees and in (A).

Identification of the SOCs above by LDna depends on the particular data set and requires the choice of values for two key parameters: |*E*|_min_ (the minimum number of edges required for a cluster to be considered) and *φ* (which controls when clusters are defined as outliers). To test the extent to which identification of the SOCs associated with inversions above depends on the choice of |*E*|_min_ and *φ*, we repeated the above LDna analyses with a wide range of parameter value combinations. Details of the resulting SOC losses and gains are shown in Fig.6A. Two of the SOCs (1128_0.27 and 840_0.43) were recovered from all of this parameter space. All the five SOCs associated with inversions and no alternative SOCs were recovered from a substantial region of parameter space (white area in Fig.6A).

Figure6B shows trees resulting from particular combinations of parameter values. Tree 1 (where *φ* = 7 and |*E*|_min_ = 20) serves as a reference point, corresponding to the tree used in the analyses above (Fig.3A). There were three main reasons why a SOC in Tree 1 was not identified when using different parameter combinations. First, when |*E*|_min_ is high, it can exceed the number of edges (|*E*|) for the cluster in question. For Tree 2, in Fig.6B (*φ* = 7, |*E*|_min_ = 70), SOC 739_0.49 is lost for this reason. Second, when *φ* was high, the associated *λ*_lim_ can exceed the *λ* value of the SOC in question. For Tree 3 (*φ* = 10, |*E*|_min_ = 20), SOC 638_0.56 is lost for this reason. Third, when *φ* was low, the identification of additional SOCs meant that a cluster appeared to be a compound of more than one outlier cluster (COC, see above). For instance, as shown in Tree 4, when *φ* = 5 (|*E*|_min_ = 20), the additional identification of SOC 777_0.47 meant that SOC 927_0.38 was not identified. Conversely, gains of SOCs tend to occur at reduced values of both parameters (the green area in A). For instance, as shown in Tree 5 where |*E*|_min_ = 5 and *φ* = 5, an additional small SOC was identified (390_0.79). Only when both parameter values were reduced to very low levels, were many additional and potentially spurious SOCs gained (Tree 6). Thus, while it is important to note that changes in |*E*|_min_ and *φ* can lead to different SOCs being identified, all the SOCs identified as corresponding to inversions were to a large extent robust to changes in these parameters.

Identification of the SOCs above by LDna could also depend on size of the data set, as clusters of loci truly sharing high LD will have fewer representatives in a data set of reduced size. To explore the effect of data set size, we carried out LDna on subsamples of the *A. baimaii* RAD sequence data set. We compared each SOC identified in the subsampled data sets to the five SOCs corresponding to inversions, here denoted ‘reference SOCs’. We subsampled at random without replacement 50% (*n* = 1914) or 25% (*n* = 957) of all the available SNPs from the full data set and analysed ten replicates each. The parameter values used were as follows: |*E*|_min_ = 16 and *φ* = 3 for the 50% subsampled data sets; and |*E*|_min_ = 14 and *φ* = 2 for the 25% subsampled data sets. These parameter values were chosen as they gave results similar to those obtained with the full data set. In particular, *φ* was kept low enough to avoid the identification of SOCs that included loci from more than one reference SOC. From the 50% subsampled data sets, we recovered SOCs corresponding to all five reference SOCs from all replicates (Fig. S3A, Supporting Information). With 25% subsampled data sets, LDna failed to identify all the SOCs corresponding to the reference SOCs in 6 of 10 replicates (denoted by pink circles in Fig. S3B, Supporting Information). In 2 of 10 replicates, SOCs not corresponding to any reference SOC were also recovered (denoted by red circles in Fig. S3B, Supporting Information). Smaller data set sizes can therefore reduce the ability of LDna to detect biologically relevant SOCs and, in some instances, lead to the detection of spurious SOCs. Nonetheless, as sequencing throughput is typically increasing, limited data set size seems unlikely to be a major impediment to the application of LDna.

### LDna can identify loci associated with local adaptation and population-demographic history

We hypothesize that in addition to inversions, LDna can be used to detect SOCs resulting from geographical structuring and local adaptation. To test this, we applied LDna to the three-spined stickleback (*Gasterosteus aculeatus*) system in which geographical structuring and local adaptation have been well characterized (Jones *et al*. 2012). This data set comprises SNP data from 21 genomes from multiple pairs of two highly morphologically and genetically distinct ecotypes locally adapted to marine and freshwater environments, from Pacific and Atlantic populations. Three small inversions on chromosomes I, XI and XXI that differ in their frequencies between the two ecotypes have previously been identified from this data set (Jones *et al*. 2012). Thus, in addition to finding SOCs corresponding to these inversions, we predict that LDna will identify SOCs resulting from population structure (Atlantic vs. Pacific) and local adaptation (Saltwater vs. Freshwater).

**Fig. 7:**
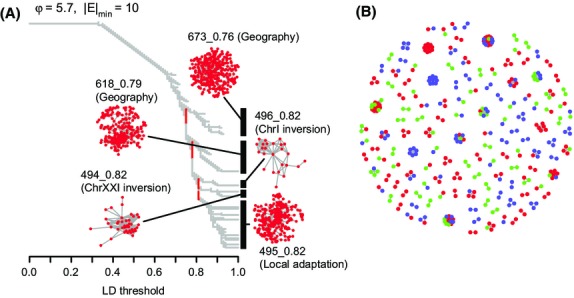
LDna on population-genomic data from the three-spined stickleback. (A) A clustering tree of pairwise LD values among 5962 SNPs from combined freshwater and marine ecotypes from the Atlantic and Pacific oceans. The data set includes only SNPs from the three chromosomes (I, XI and XXI) that contain known inversions. Clusters identified as SOCs by LDna (at the parameter values indicated in the figure) are also shown with likely evolutionary cause indicated (see main text and Fig.8 for details). (B) A full network for LD threshold = 0.95. Each locus is coloured according the chromosome to which it belongs: green, red and blue for I, XI and XXI, respectively. All large clusters (|*E*| > 10 at a threshold of 0.95) with loci from more than one chromosome are nested within SOC 495_0.82.

We applied LDna to a high-quality subset of 5962 SNPs from the chromosomes with known inversions (I, XI and XXI). Exploring variation across parameter values (as demonstrated in Fig.6 and in Appendix S1 and S2, Supporting Information) allowed us to identify five SOCs (494_0.82, 495_0.82, 496_0.82, 618_0.79 and 673_0.76; Table 1) corresponding to each of the large branches in Fig.7A at |*E*|_min_ = 10 and *φ* = 5.7. All loci from SOC 496_0.82 mapped to the chromosome I inversion, and all but four loci (4 of 41) from SOC 494_0.82 mapped to the chromosome XXI inversion. No SOCs mapped specifically to the known inversion on chromosome XI, probably because not all SNPs were used (see Appendix S4) and the inversion is small. In contrast, the three remaining SOCs contain loci widely distributed across all three chromosomes. One of these SOCs (495_0.82) contains loci across all three chromosomes in particularly high LD (>0.95, Fig.7B). Consequently, we infer that two SOCs correspond to two of the three previously identified inversions and the three remaining SOCs correspond to LD clusters arising from other causes.

**Fig. 8:**
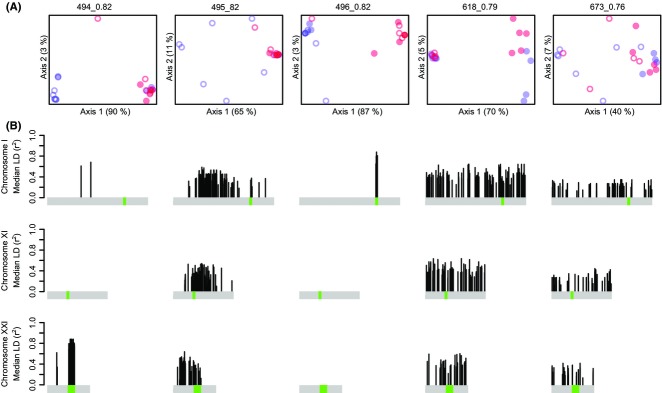
Population-genetic analyses of stickleback SOCs. (A) Population structuring of SOC loci (as identified in Fig.7A) based on the first two components from PCA. Each circle represents an individual, coloured blue for Freshwater or red for Marine environment. Open and filled circles represent Pacific or Atlantic origin, respectively. Per cent variation explained by each component is indicated along the axes. (B) Bars show the positions of SOC loci on each of the chromosomes: I, XI and XXI. Each column has loci from one SOC as labelled in part (A) above. Bar height shows the median of all intra-SOC LD values for a given locus. Green regions indicate the position of inversions on each chromosome.

The association of each SOC with respect to population structure (Atlantic vs. Pacific) and local adaptation (marine vs. freshwater) was assessed by PCA. Three of the five SOCs (494_0.82, 495_0.82 and 496_0.82), including the two that correspond to inversions, broadly separate freshwater and marine ecotypes (blue vs. red in Fig.8A). This is consistent with these SOCs comprising loci associated with adaption to freshwater or marine habitats. In the case of 495_0.82, the separation is specifically between freshwater Pacific individuals and all others. Loci from the remaining two SOCs (618_0.79 and 673_0.76) broadly separate individuals from Pacific and Atlantic populations (open vs. filled in Fig.8A). Overall, these analyses reveal that LDna can identify LD clusters associated with at least three different (and sometimes overlapping) evolutionary phenomena: inversions, local adaptation and geographical population structure.

## Discussion

Here, we have developed and used LDna to detect multiple linked and unlinked subsets of loci sharing high LD. Analyses of these subsets of loci using a range of population-genetic analyses then enabled us to infer how they are involved in different evolutionary phenomena: inversions, local adaptation and geographical structure. Below we discuss the empirical findings, before turning to the usefulness of LDna in the context of other methods available to study genomewide LD.

### LDna and inversions

Through their effect on inhibiting recombination, inversions play an important role in evolution, particularly in local adaptation and speciation (Kirkpatrick & Barton 2006; Hoffmann & Rieseberg 2008; Lowry & Willis 2010). Traditionally, studying inversions required cytological studies (e.g. fluorescence *in situ* hybridization techniques; Tang *et al*. 2008), BAC-clone sequencing (Tang *et al*. 2008) and/or sequencing of full genomes (Corbett-Detig *et al*. 2012). These are laborious and/or expensive, particularly in nonmodel species. Here, we demonstrated that LDna, coupled with population-genetic analyses, can be used to identify loci putatively associated with inversions in both a timely and cost-effective manner, even without mapping information. Such inversions can be both large, as in *Anopheles baimaii*, and small, as in the sticklebacks. Further, if there are SNPs within SOCs that are fixed (or almost fixed) between the inversion karyotypes, these could potentially be used as inversion markers to facilitate large-scale studies of inversion polymorphism in natural populations. Thus, LDna opens up the possibility of studying inversion polymorphism, by relatively simple means, in any species for which a population-genomic data set can be generated.

### LDna and local adaptation

In the original generation and analysis of the stickleback data set used here, Jones *et al*. (2012) used supervised approaches to identify a large number of genomic regions that were consistently associated with marine–freshwater divergence. In contrast, LDna allows an unsupervised approach to detect clusters of loci in high LD across the whole genome, from any source in a single analysis. Contrary to what might have been expected from the original study, we did not find a unique SOC that separated marine and freshwater individuals globally (i.e. regardless of which ocean they were sampled from). Instead, we found one SOC (495_0.82) associated with adaptation to freshwater in the Pacific only. It is thus possible that a large part of the divergence between marine and freshwater ecotypes observed in the original study is driven by differences specifically between the ecotypes in the Pacific. Such unexpected patterns may be difficult to detect by supervised approaches (in which groups between which differences are sought need to be defined a priori) including standard divergence-based outlier analyses. LDna, as an unsupervised approach, can therefore provide a more nuanced view of loci involved in complex adaptations.

There are several distinct subclusters visible within SOC 495_0.82 (Fig.7A), comprised of a surprisingly large number of loci spread across all the three chromosomes analysed here (Fig.7B). It is likely that only a few loci in SOC 495_0.82 are directly involved in local adaptation (either due to selection acting in parallel in different freshwater systems or epistatic fitness interactions; Hohenlohe *et al*. 2012). Instead, the large number of loci in this SOC likely result from divergence hitchhiking (Via 2011) coupled with the reduced effects of recombination due to geographical structuring. Loci within a SOC that are not physically colocated can provide good candidates for loci directly associated with parallel selection or epistatic fitness interactions. These include the individual loci in exceptionally high LD across chromosomes as indicated by clusters with a mix of loci from different chromosomes in Fig.7B. The four loci in the SOC associated with the chromosome XXI inversion (494_0.82) that map outside it are good candidates. In particular, the one with the highest LD to the rest of the cluster falls within the predicted gene ENSGACT00000014703 on chromosome I, encoding a protein homologous to the dynein light chain, involved in intracellular vesicle transport. This gene is known to be significantly associated with marine–freshwater divergence (it has a colocated peak in the ‘Marine-Freshwater Cluster Separation Score’, one of 174 with a genomewide false discovery rate of *P* < 0.05; Jones *et al*. 2012).

### LDna and geographical structure

We found two SOCs (618_0.79 and 673_0.76) associated with Atlantic–Pacific structuring in the sticklebacks. Closer examination of the allele frequencies at these loci (Fig. S4) shows highly contrasting patterns. For SOC 673_0.76, many loci that are heterozygous in the Pacific are homozygous in the Atlantic. This is consistent with a founder event following the spread of this species from the Pacific to the Atlantic (Colosimo *et al*. 2005), with the associated drift resulting in the loss of genetic diversity in the Atlantic population. In contrast, in SOC 618_0.79, the allele frequency differences are far more divergent between the oceans (*F*_ST_ = 0.64 vs. 0.10 for SOC 673_0.76). In other words, this SOC comprises the most differentiated loci between the oceans – those that are either fixed or nearly fixed between them (Fig. S4). Interestingly, within 618_0.79, the PCA also identified some differentiation between freshwater and marine environments for Atlantic individuals (Fig.8A) indicating that some of these loci may also be involved in marine–freshwater divergence, specifically within the Atlantic. Overall, this demonstrates that LDna can separate different evolutionary phenomena even when they are associated with the same historical separation event.

### Approaches to the study of genomewide LD

Typically, LD declines quickly over short physical distances in wild populations (Kim *et al*. 2007; Slate & Pemberton 2007; Gray *et al*. 2009). Despite this, LD can span large contiguous genomic regions within chromosomes, as has been well documented in humans (e.g. Conrad *et al*. 2006). Several methods have been developed to characterize and utilize this information on LD. These include the integrated haplotype score (iHS) test (Voight *et al*. 2006) and the cross-population extended haplotype homozygosity (XP-EHH) test (Sabeti *et al*. 2007) that detect extended haplotypes that indicate the action of natural selection. Other methods have accessed such information on haplotypes and correlated allele frequencies to increase the power to make inferences of population structure, admixture and demography (Falush *et al*. 2003; Lawson *et al*. 2012; Ralph & Coop 2013).

It is becoming increasingly clear that LD can also occur among noncontiguous regions of the genome, even between chromosomes, in many taxa including humans (Wilson & Goldstein 2000; Hohenlohe *et al*. 2012; Koch *et al*. 2013; Schumer *et al*. 2014). Approaches to understand cross-genome (rather than localized) patterns of LD tend to focus on pairwise comparisons between loci/haplotype blocks. While the LDna approach also relies on a matrix of pairwise estimates of LD, its use of networks goes beyond pairwise comparisons to identify sets of loci sharing high LD. This potentially enables LDna to capture information about high-order LD within the genome.

## Conclusions

The insights provided by LDna are possible in any population-genomic data set, but are likely to be particularly valuable for nonmodel species where a global view of the genomic architecture is otherwise difficult to gain. We were able not only to detect potentially unexpected signals of LD (such as those caused by inversions), but to partition loci into sets affected by different evolutionary phenomena. This gives confidence that LDna will also provide insights in other situations where a complex LD signal involving noncontiguous parts of the genome is expected (e.g. assortative mating, epistatic interactions among multiple loci and species introgression). LDna could also be used to separate clusters of loci in high LD with the purpose of removing ‘outliers’ prior to studies that require neutral markers, for example to estimate population structure and population history. This broad applicability is coupled with access to a global view of evolutionary phenomena affecting genomes and the possibility of reasoned partitioning of loci within them, without prior assumptions. Together, these features make LDna an excellent exploratory tool for any population-genomic data set.

## Data accessibility

LDna is implemented as a package in r (R Core Team 2014) and is freely available from https://github.com/petrikemppainen/LDna. All data sets used in this study and R-scripts to reproduced analyses and figures presented here are available at Dryad: http://doi.org/10.5061/dryad.2t764.

## Supporting Information

**Fig. S1** Map of geographical sampling locations for *Anopheles baimaii*.

**Fig. S2** Bayesian information criterion vs. number of groups for *Anopheles baimaii* SOCs.

**Fig. S3** The effect of numbers of loci on LDna for *Anopheles baimaii*.

**Fig. S4** Genotype differences between Atlantic and Pacific populations for SOCs 618_0.79 and 673_0.76.

**Appendix S1** An introduction to LDna: basics. Tutorial which gives an introduction to the r package ‘LDna’. A continually updated version can be found at: https://github.com/petrikemppainen/LDna

**Appendix S2** An introduction to LDna: advanced. Tutorial which explains some of the more advanced features of LDna including suggestions on how to find appropriate values for the parameters *φ* and |*E*|_min_. A continually updated version is available from: https://github.com/petrikemppainen/LDna

**Appendix S3** Linkage disequilibrium network analysis (LDna) on simulated data

**Appendix S4** *Anopheles baimaii* RAD sequences data set and three-spined sticklebacks SNPs data set preparation

**Appendix S5** *Anopheles baimaii* linkage map preparation
