# Supplementary material for: Linkage disequilibrium network analysis (LDna) gives a global view of chromosomal inversions, local adaptation and geographic structure
Source: Mol Ecol Resour. 2015 Jan 21;15(5):1031–45. doi: 10.1111/1755-0998.12369 (PMC4681347; doi:10.1111/1755-0998.12369)
Supplement: Supplementary file 1 — Fig. S1 Map of geographical sampling locations for Anopheles baimaii. [file men0015-1031-sd1.pdf]

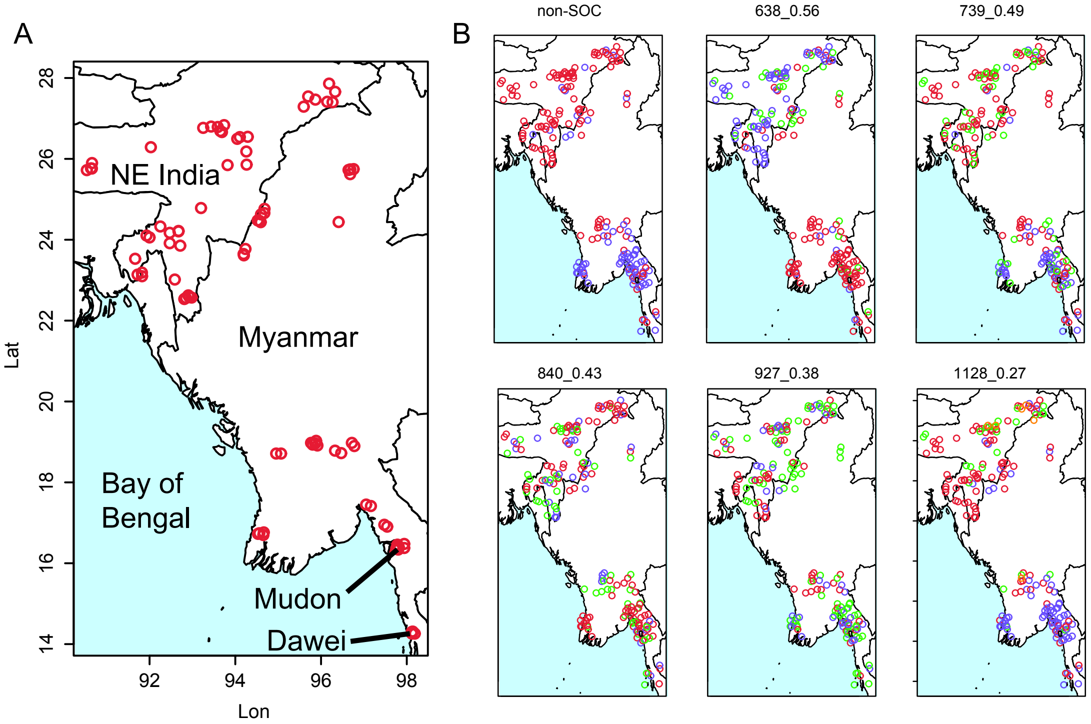

Fig. S1 Sampling locations and the distribution of inferred karyotypes. (A) Shows the 91 unique sampling locations. Mudon and Dawai indicate locations where P1 individuals for the linkage map cross came from (see Appendix S5, Supporting Information). (B) Shows the geographic distribution of inferred karyotypes for each SOC, with individuals colored according to in Fig. 4, main text. Some noise has been added to the coordinates to make individuals more visible.
