# Supplementary material for: Linkage disequilibrium network analysis (LDna) gives a global view of chromosomal inversions, local adaptation and geographic structure
Source: Mol Ecol Resour. 2015 Jan 21;15(5):1031–45. doi: 10.1111/1755-0998.12369 (PMC4681347; doi:10.1111/1755-0998.12369)
Supplement: Supplementary file 2 — Fig. S2 Bayesian information criterion vs. number of groups for Anopheles baimaii SOCs. [file men0015-1031-sd2.pdf]

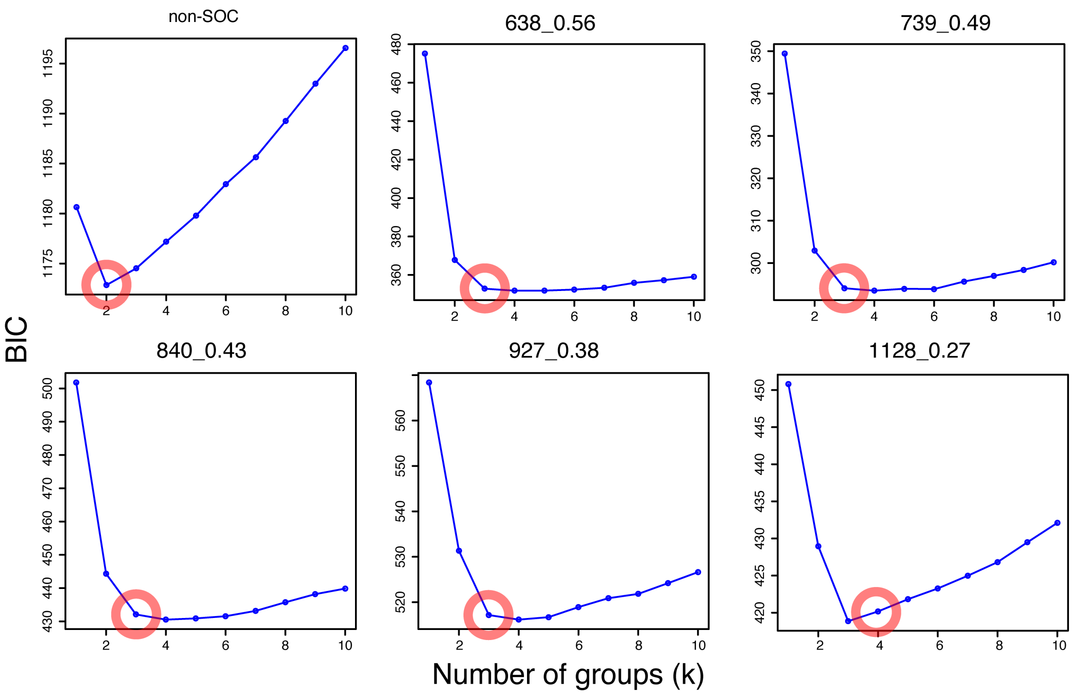

Fig. S2 Bayesian Information Criterion (BIC) versus number of groups for *A. baimaii* SOCs. Circles indicate the 'elbows' of these relationships based on which the clusters solution for each set of loci were chosen (see *Adegenet* documentation for details referenced in Materials and Methods). For SOC 1128_0.27 four groups were chosen as this solution improved the partitioning of individuals according to karyotype (see main text for details).
