# Supplementary material for: Linkage disequilibrium network analysis (LDna) gives a global view of chromosomal inversions, local adaptation and geographic structure
Source: Mol Ecol Resour. 2015 Jan 21;15(5):1031–45. doi: 10.1111/1755-0998.12369 (PMC4681347; doi:10.1111/1755-0998.12369)
Supplement: Supplementary file 3 — Fig. S3 The effect of numbers of loci on LDna for Anopheles baimaii. [file men0015-1031-sd3.pdf]

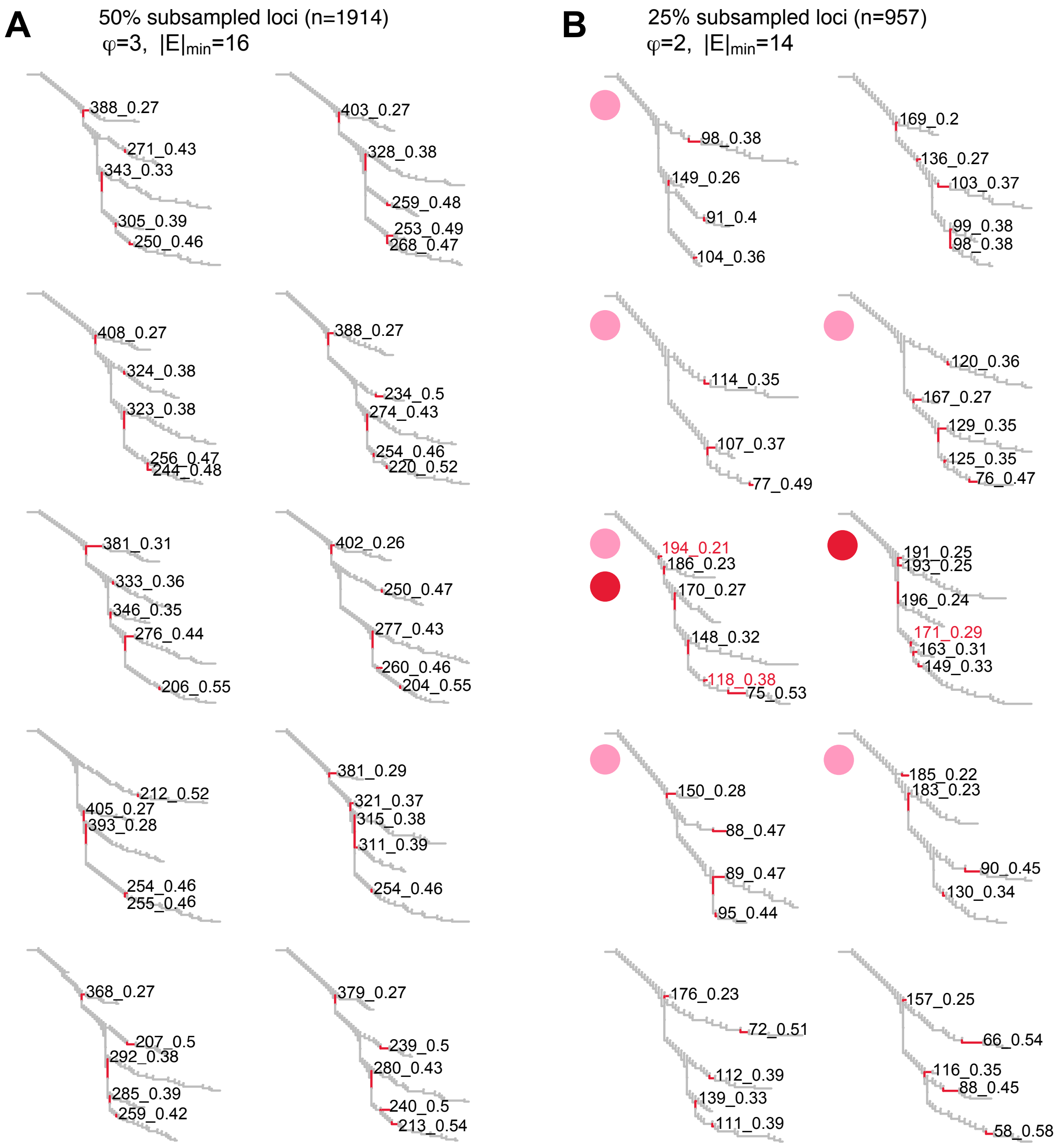

Fig. S3 Shows the effect of numbers of loci on LDna. Linkage disequilibrium network analysis was repeated (*n*=10) on data sets comprising subsamples of the original *A. baimaii* RAD sequence data. (A) and (B) shows results for 50% and 25% subsampled loci respectively with parameter values for φ and $|E|_{min}$ indicated above each figure. SOCs from each replicate were compared to the reference SOCs (results from LDna of the full data set, see main text for details). Pink color indicates SOC loss, i.e. an LDna result where no SOC contained loci from at least one of the five reference SOCs. Red color indicates SOC gain, i.e. results where at least one SOC did not contain any loci from any of the reference SOCs.
