# Supplementary material for: Linkage disequilibrium network analysis (LDna) gives a global view of chromosomal inversions, local adaptation and geographic structure
Source: Mol Ecol Resour. 2015 Jan 21;15(5):1031–45. doi: 10.1111/1755-0998.12369 (PMC4681347; doi:10.1111/1755-0998.12369)
Supplement: Supplementary file 4 — Fig. S4 Genotype differences between Atlantic and Pacific populations for SOCs 618_0.79 and 673_0.76. [file men0015-1031-sd4.pdf]

A

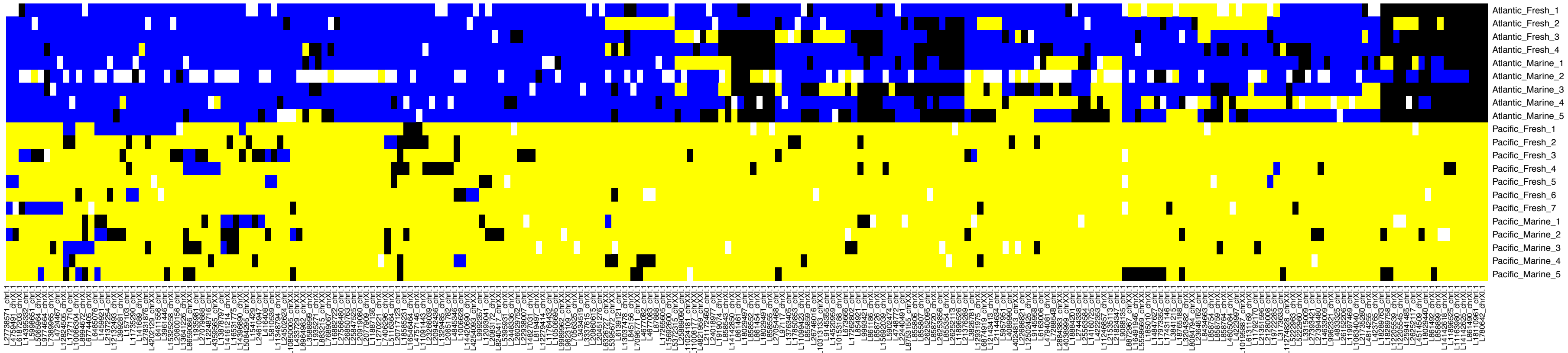

B

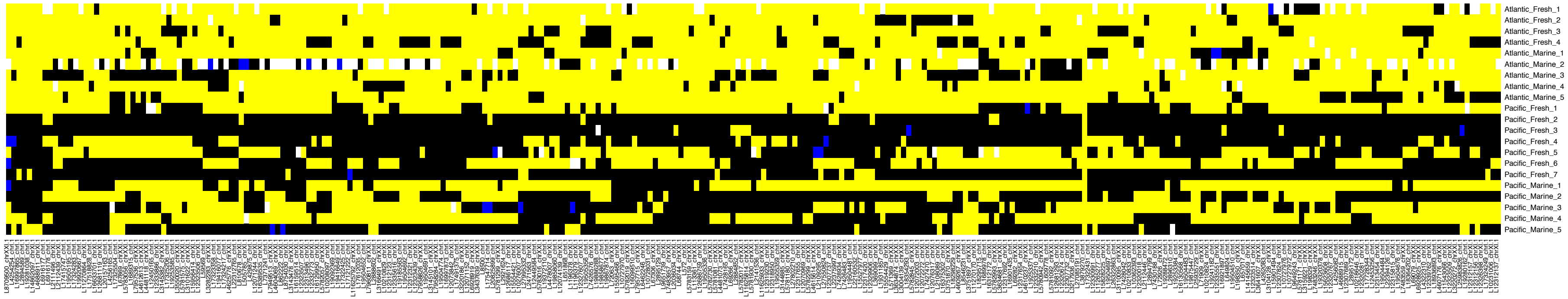

Fig. S4 Genotype differences between Atlantic and Pacific populations for SOCs 618_0.79 and 673_0.76. Shows raw genotypes for loci in SOCs 618_0.79 (A) and 673_0.76 (B) where blue is homozygote for the most common SNP in the Atlantic, yellow is homozygote for the most common allele in the Pacific black is heterozygote and white indicates missing data.
