# Supplementary material for: Linkage disequilibrium network analysis (LDna) gives a global view of chromosomal inversions, local adaptation and geographic structure
Source: Mol Ecol Resour. 2015 Jan 21;15(5):1031–45. doi: 10.1111/1755-0998.12369 (PMC4681347; doi:10.1111/1755-0998.12369)
Supplement: Supplementary file 5 — Appendix S1 An introduction to LDna: basics. Tutorial which gives an introduction to the r package ‘LDna’. A continually updated version can be found at: https://github.com/petrikemppainen/LDna [file men0015-1031-sd5.pdf]

An introduction LDna: Basics

Petri kemppainen, October 7, 2014

Introduction

This vignette explains the principle of linkage disequilibrium network analyses (LDna) using the R-package **LDna**. LDna uses network analyses to find clusters of loci in high linkage disequilibrium (LD) from population genomic data sets. In LD networks vertices represent loci and edges represent LD values above given thresholds. Starting from a high LD threshold value, only few loci will be connected to each other. As the LD threshold is lowered, more and more edges and loci are added to the network allowing clusters to 'grow' with respect to numbers of loci and edges. Distinct clusters that remain separate across large range of LD thresholds indicate subsets of loci that represent different population genetic signals in the data. At sufficiently low LD threshold values all loci will eventually merge (connect by at least one edge) into a single cluster. Trees are used to visualise the process of cluster merging with decreasing LD threshold. This is *single linkage clustering* (see `?hclust` for details) as only one connection (edge) between clusters is required for them to be considered as 'merged'. In these trees nodes represent the merger of clusters and distance to nodes the LD threshold values at which clusters merge.

Linkage disequilibrium network analysis relies on the premise that 'interesting' clusters remain distinct (i.e. separate) across a wide range of LD thresholds and can be identified/extracted for downstream analyses at an LD threshold value just before they merge. To identify these mergers we record the change in the median LD between all loci in a cluster before and after merger, λ. This is defined as: $\left(\widetilde{x}_1 - \widetilde{x}_2\right)n$ where $\widetilde{x}_1$ is the median of all pairwise intra-cluster LD values within the focal cluster, $\widetilde{x}_2$ the median of all pairwise intra-cluster LD values after merger and n is the number of loci in the focal cluster. Thus, the addition of individual loci to an existing cluster or the merging of clusters where only one cluster is large (with respect to numbers of loci) will cause small λ-values. In contrast, the merging of at least two large and distinct clusters with highly different LD signals will cause high λ-values for each of the large clusters involved. Clusters with λ-values that comprise outliers (relative to the median of all values in the data set) are then visually explored and subsequently extracted for downstream analyses.

Installing

With **devtools** (accessible from CRAN) the R-package **LDna** can be installed by:

```
devtools::install_github("petrikemppainen/LDna")
```

This downloads the source directly from **github** and builds the vignettes and thus requires LaTeX to be installed on your computer.

Alternatively, download the source file from the LDna github repository an install by:

```
install.packages("/path_to/source_file", repos = NULL, type = "source")
```

and then load the package:

```
library(LDna)
```

Linkage disequilibrium network analysis

About estimating LD

The only input data for **LDna** is an upper or lower matrix of all pairwise LD values between loci from a population genomics data set. Calculating these values is the primary bottleneck of LDna analyses as the number of pairwise comparisons increase by $n*(n-1)/2$ where n is the number of loci in the data set. Although not the fastest method, LD can be estimated by the function `LD` from the **genetics** package (see `?genetics::LD` for details). The squared correlation coefficient between pairs of loci, r^2, is strongly recommended as it takes into account the heterozygosity of the loci (which other common estimates of LD, such as D or D', do not). Note that r^2 can only be calculated for bi-allelic markers.

Produce raw data for LDna

Sample data set Although LDna analyses deal with large population genomic data sets, here we show the principles of LDna using an LD matrix with only eight loci.

```
LDmat <- structure(c(NA, 0.84, 0.64, 0.24, 0.2, 0.16, 0.44, 0.44, NA, NA, 0.8,
    0.28, 0.4, 0.36, 0.36, 0.24, NA, NA, NA, 0.48, 0.32, 0.2, 0.36, 0.2, NA,
    NA, NA, NA, 0.76, 0.56, 0.6, 0.2, NA, NA, NA, NA, NA, 0.72, 0.68, 0.24,
    NA, NA, NA, NA, NA, NA, 0.44, 0.24, NA, NA, NA, NA, NA, NA, NA, 0.2, NA,
    NA, NA, NA, NA, NA, NA, NA), .Dim = c(8L, 8L), .Dimnames = list(paste("L",
    1:8, sep = ""), paste("L", 1:8, sep = "")))
```

```
LDmat
```

```
     L1   L2   L3   L4   L5   L6  L7 L8
L1   NA   NA   NA   NA   NA   NA  NA NA
L2 0.84   NA   NA   NA   NA   NA  NA NA
L3 0.64 0.80   NA   NA   NA   NA  NA NA
L4 0.24 0.28 0.48   NA   NA   NA  NA NA
L5 0.20 0.40 0.32 0.76   NA   NA  NA NA
L6 0.16 0.36 0.20 0.56 0.72   NA  NA NA
L7 0.44 0.36 0.36 0.60 0.68 0.44  NA NA
L8 0.44 0.24 0.20 0.20 0.24 0.24 0.2 NA
```

The first step in LDna is to build a *single linkage clustering* tree from an LD matrix and acquire summary data for each of the clusters identified by this tree. This is achieved by the function `LDnaRaw` which takes an upper or lower diagonal LD matrix and outputs a list with two objects. The first, `ldna$clusterfile`, is a matrix with all unique clusters as columns and the loci as rows, where `TRUE` indicates the presence of a locus in a specific cluster (else `FALSE` is given). The second, `ldna$stats`, is a data frame that gives all edges for the above tree along with summary information, including λ. By default all LD values are rounded to the nearest hundred as this significantly reduces computation time and has little affect on the analyses as a whole.

```
ldna <- LDnaRaw(LDmat)
```

As seen from the object `ldna$clusterfile` this data set includes seven unique clusters of which *"7_0.44"* includes all loci. The number after the underscore for each cluster-name indicates the highest LD threshold at which it is present.

```
ldna$clusterfile
```

```
   7_0.44 6_0.48 2_0.8 5_0.68 4_0.72 3_0.76 1_0.84
L1   TRUE   TRUE  TRUE  FALSE  FALSE  FALSE   TRUE
L2   TRUE   TRUE  TRUE  FALSE  FALSE  FALSE   TRUE
L3   TRUE   TRUE  TRUE  FALSE  FALSE  FALSE  FALSE
L4   TRUE   TRUE FALSE   TRUE   TRUE   TRUE  FALSE
L5   TRUE   TRUE FALSE   TRUE   TRUE   TRUE  FALSE
L6   TRUE   TRUE FALSE   TRUE   TRUE  FALSE  FALSE
L7   TRUE   TRUE FALSE   TRUE  FALSE  FALSE  FALSE
L8   TRUE  FALSE FALSE  FALSE  FALSE  FALSE  FALSE
```

In the object `ldna$stats` each row represents a cluster with the following information for each: name for the focal cluster, name for the parent cluster (cluster after merger), distance between nodes from when a cluster first appears until it merges again (in units of LD), the number of edges, nE, number of vertices (loci), nV, and λ.

```
ldna$stats
```

```
   cluster parent_cluster distance nV nE lambda
1   7_0.44           root     0.44  8 12   0.00
2   6_0.48         7_0.44     0.04  7  9   0.56
3   5_0.68         6_0.48      0.2  4  3   1.04
4   4_0.72         5_0.68     0.04  3  2   0.24
5    2_0.8         6_0.48     0.32  3  2   1.32
6   3_0.76         4_0.72     0.04  2  1   0.08
7   1_0.84          2_0.8     0.04  2  1   0.08
8       L5         3_0.76     0.24  1  0   0.00
9       L4         3_0.76     0.24  1  0   0.00
10      L6         4_0.72     0.28  1  0   0.00
11      L7         5_0.68     0.32  1  0   0.00
12      L2         1_0.84     0.16  1  0   0.00
13      L1         1_0.84     0.16  1  0   0.00
14      L3          2_0.8      0.2  1  0   0.00
15      L8         7_0.44     0.56  1  0   0.00
```

Identify and extract clusters

Next we proceed with studying the tree and extracting clusters using the function `extractClusters`. In this process we need to decide which clusters are consider as outliers with respect to their λ-values. An outlier cluster (OC) is defined as any cluster with a λ-value higher than

$$\lambda_{lim(\varphi)} = median + mad * \varphi$$

where mad is the *median absolute deviation* (see `?mad` for details) across all λ-values, and φ is a user defined factor (the argument `constant` for the `mad` function). Thus, φ (controlled by the argument `phi`) determines how stringent LDna is with respect to considering a cluster an OC. This makes the absolute values of λ irrelevant as each value is assessed in relation to all other values in the data set/tree. It is also possible to define the minimum number of edges, $|E|_{min}$ (specified by the argument `min.edges`), to restrict which branches are shown in the tree (this is necessary for larger data sets and will be covered in more detail in the `LDna_advanced` vignette, accessed through: `vignette("LDna_advanced", package="LDna")`). Here we set `min.edges=0` in order to visualise the entire tree, including branches representing individual loci. We set `phi=1`.

By default, the function `extractClusters` plots two figures. The first shows all λ-values in increasing order, where the red dashed line indicates λ_{lim} above which any value is considered as an outlier (indicated in red). In this analysis two clusters: *2_08* and *5_58*, are outliers. The second figure shows the tree that visualises the cluster merging process. Only tip labels for the two internal branches that comprise *OCs* are shown and the branches leading to these nodes are colored red. Note also the extra tips with zero branch lengths associated with every node. These are added in order to access the nodes as tips by functions from the **ape** package.

```r
par(mfcol=c(1,2))
clusters1 <- extractClusters(ldna, min.edges = 0, phi = 1)
```

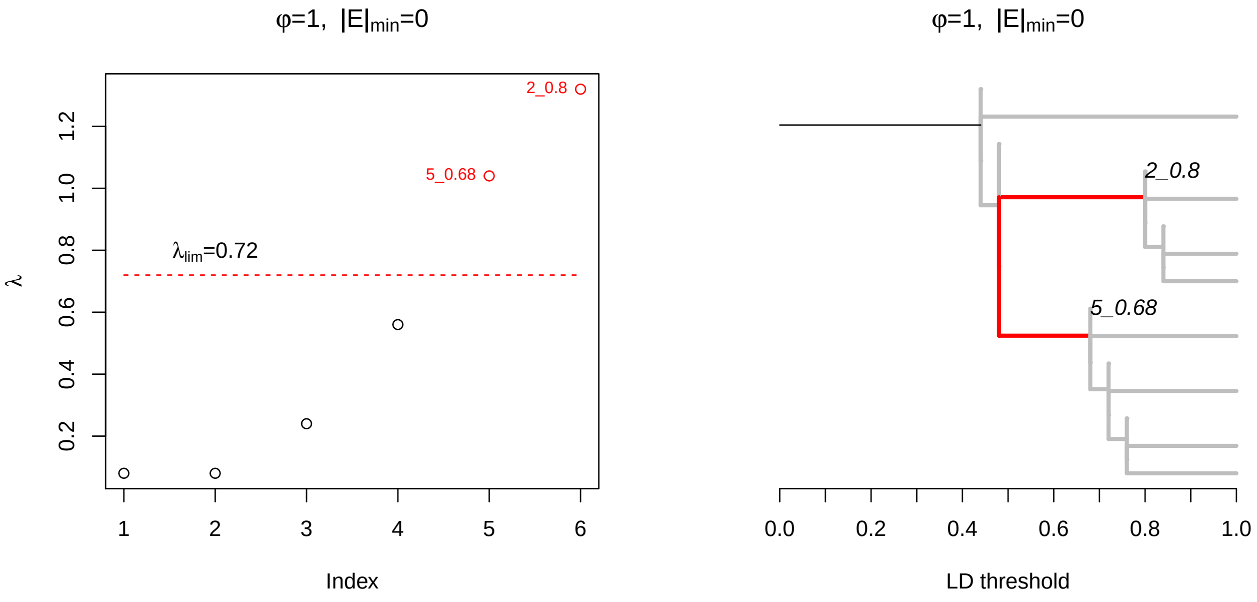

`extractClusters` outputs a list with all OCs and their loci.

```r
clusters1
```

```
$`2_0.8`
[1] "L1" "L2" "L3"

$`5_0.68`
[1] "L4" "L5" "L6" "L7"
```

If `phi` is lowered to 0.25 one additional cluster, *6_0.48*, becomes an outlier.

```r
par(mfcol=c(1,2))
clusters2 <- extractClusters(ldna, min.edges = 0, phi = 0.25, rm.COCs=FALSE)
```

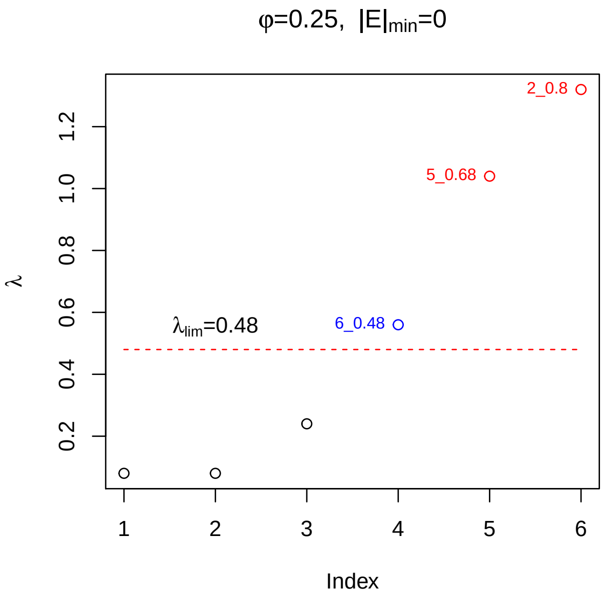

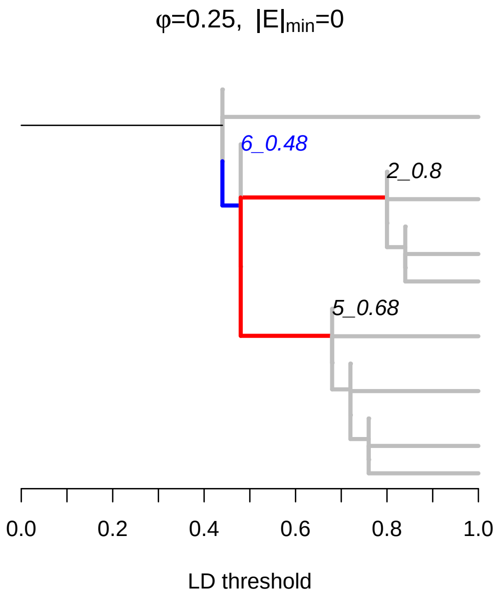


```r
clusters2
```

```
$`2_0.8`
[1] "L1" "L2" "L3"

$`5_0.68`
[1] "L4" "L5" "L6" "L7"

$`6_0.48`
[1] "L1" "L2" "L3" "L4" "L5" "L6" "L7"
```

As seen above, *OC 6_0.48* represents the merging of the two previous *OCs* (*2_0.8* and *5_0.68*). To distinguish that an *OC* contains loci from those extracted at an higher LD threshold than itself we denote it as a *"compound outlier cluster"*, *"COC"*, as opposed to a *"single outlier cluster"*, *"SOC"*. Typically we are mainly interested in *SOCs* and thus by default only *SOCs* are considered. However, as in the example above, when `rm.COCs=FALSE` all *OCs* are shown in the figures (and extracted) and any *COCs* are colored blue.

Summerise data

Based on the data produced above (`ldna`, `clusters` and `LDmat`), *OCs* can be further summarized by the function `summaryLDna`. In addition to the information already available from the `ldna` file above, `summaryLDna` outputs a data frame with the following information for each extracted cluster: *"Type"*, which indicates whether an *OC* is a *SOC* or a *COC* (for the given setting for `min.edges` and `phi`); *"Merge.at"*, which indicates at which LD threshold the cluster merges with the next cluster; *"Median.LD"* which gives the median of all intra-cluster pairwise LD values and *"MAD.LD"* which gives the unscaled median abosulute deviation (see `?mad`) of these LD values. A large difference between the LD threshold at which a cluster first is present (as indicated by the cluster name, see above) and *"Merge.at"* indicates that the cluster remains the same (with respect to it's loci) for a large range of LD threshold values.

```r
summary <- summaryLDna(ldna, clusters2, LDmat)
summary
```

```
       Type Merge.at nLoci nE lambda Median.LD MAD.LD
```

2_0.8	SOC	0.48	3	2	1.32	0.80	0.04
5_0.68	SOC	0.48	4	3	1.04	0.64	0.08
6_0.48	COC	0.44	7	9	0.56	0.44	0.16

Visual inspection of networks

Lastly the function `plotLDcluster` is used to visually inspect the extracted clusters. This function is based on functions from the package **igraph** and always also needs the original LD matrix as input. First we can plot the full network at a given threshold by setting `option=1` and specifying the threshold. This can be done without any additional information besides the LD matrix and gives an instant impression of how much clustering exists in the data. Thus, when exploring new data sets this should be the first analysis to do, see `LDna_advanced` for details. At LD `threshold=0.6` the two *OCs* (corresponding to *2_0.08* and *5_0.68*) are still separate, i.e. not connect by any edge.

```
plotLDnetwork(LDmat=LDmat, option=1, threshold=0.6)
```

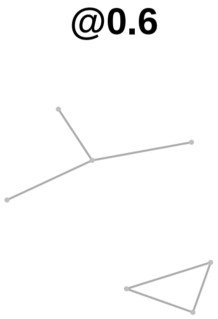

At LD threshold=0.4 all loci are connected to a single cluster by at least one edge. If the LD threshold is lowered even further more edges will be added to the network but the loci will remain the same (a cluster is defined by its unique set of loci not its set of edges).

```
plotLDnetwork(LDmat=LDmat, option=1, threshold=0.40)
```

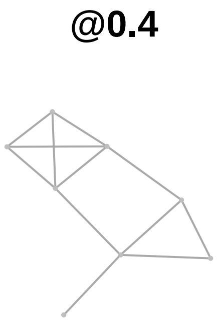

At the core `extractClusters` lie two functions from the package **igraph**: `graph.adjacency` and `plot.igraph`. The below example is used to show how these functions can be used to produce custom networks, in our case to show the locus names as well as the LD values connecting each of these loci. **Igraph** is highly flexible and `?igraph` is a good place to start.

```
# Creates a 'weighted' graph object from our lower diagonal LD matrix
g <- graph.adjacency(LDmat, mode="lower", diag=FALSE, weighted=T)
# Removes edges with weights (LD values) below 0.5
g <- delete.edges(g, which(E(g)$weight<=0.5))
# Removes 'unconnected' vertices (loci)
```

```r
g <- delete.vertices(g, which(degree(g) < 1))
# plots graph, 'edge.label' is used to add the LD values for each edge
plot.igraph(g, edge.label=E(g)$weight)
```

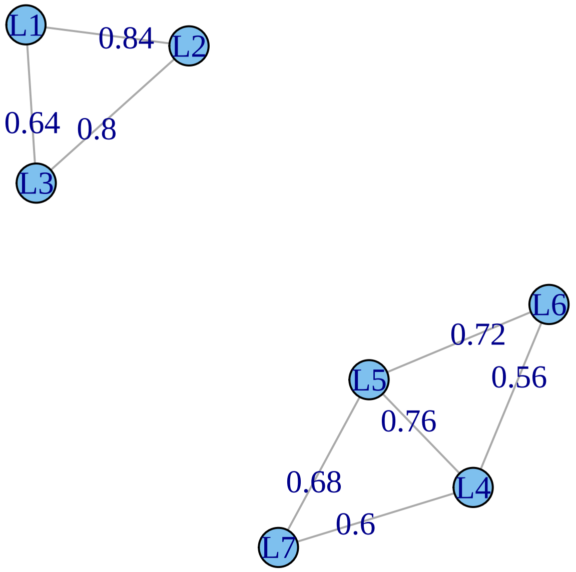

With `option=2`, by default `plotLDnetwork` plots a network for each *OC* at an LD threshold just before it merges. For this the outputs from `LDnaRaw` and `extractClusters` and `summaryLDna` also need to be included. The title gives the cluster name in question and the LD threshold at which the network is drawn and any loci from the *OC* in question is indicated in red (*SOC*) or blue (*COC*).

```r
par(mfcol=c(1,3))
plotLDnetwork(ldna, LDmat, option=2, clusters=clusters2, summary=summary)
```

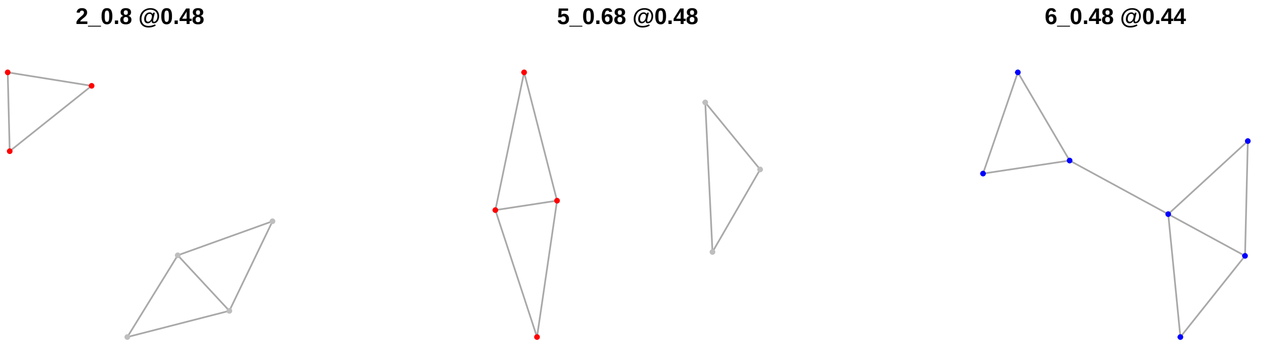

By setting the argument `after.merger` to `TRUE`, the network is printed at an LD threshold just after merger. Note thus that the networks (below) are plotted at different LD thresholds compared to above. Overall `extractClusters` is a highly customizable functions which will be further demonstrated in `LDna:advanced`.

```r
par(mfcol=c(1,3))
plotLDnetwork(ldna, LDmat, option=2, clusters=clusters2, summary=summary, after.merger=TRUE)
```

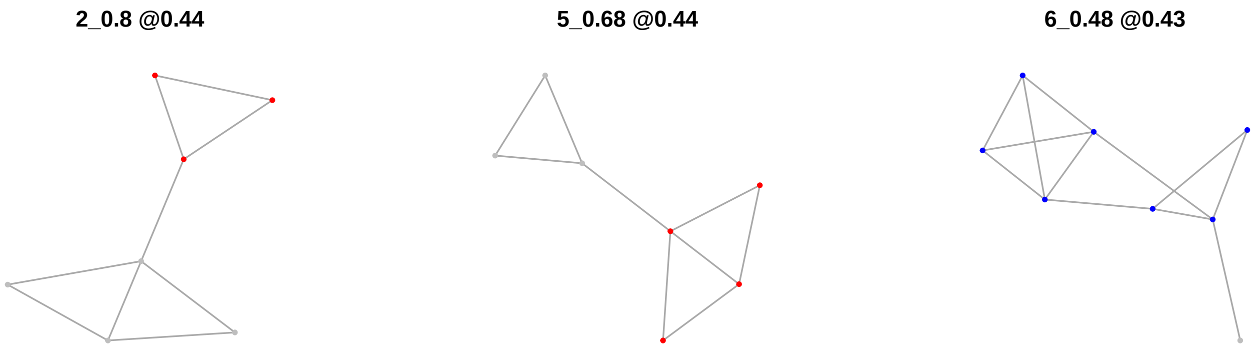

Lastly, there is also a possibility to use a fixed value of λ_{lim} via the `lambda.lim` option in `excractClusters` as follows:

```
clusters3 <- extractClusters(ldna, min.edges = 0, lambda.lim = 0.5, rm.COCs = FALSE)
```

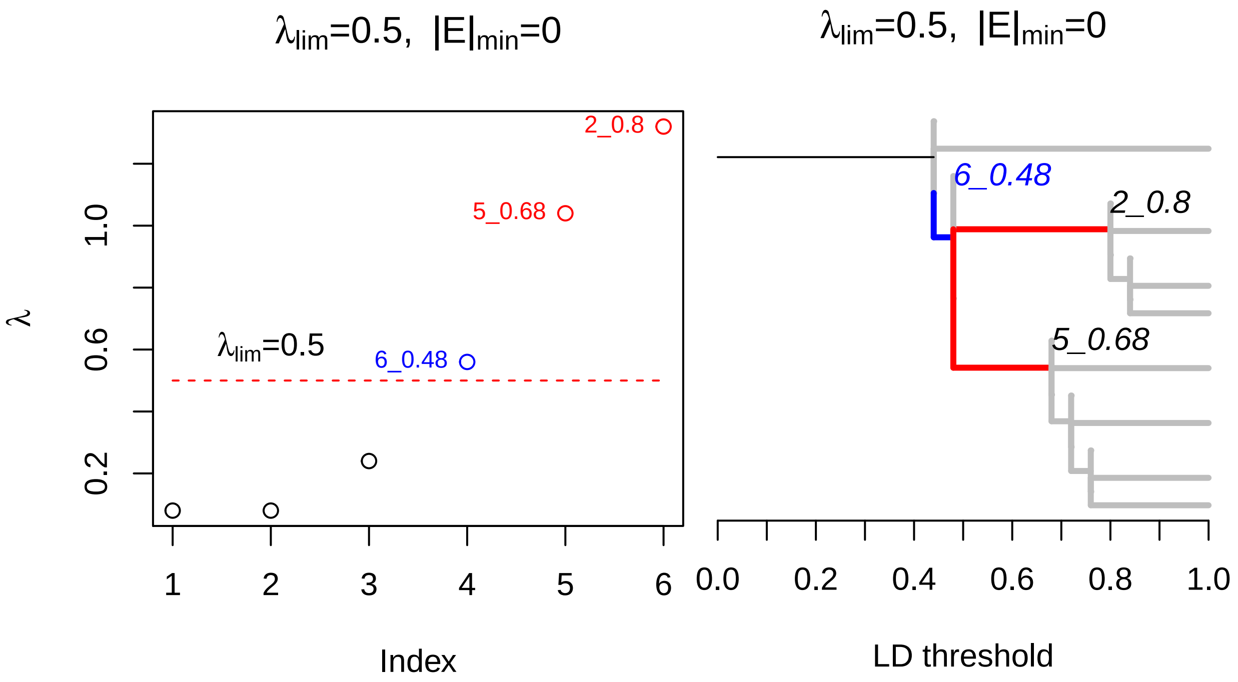

This will gives the same outcome as having `phi=0.25` (as this gives λ_{lim}=0.48)
