# Supplementary material for: Linkage disequilibrium network analysis (LDna) gives a global view of chromosomal inversions, local adaptation and geographic structure
Source: Mol Ecol Resour. 2015 Jan 21;15(5):1031–45. doi: 10.1111/1755-0998.12369 (PMC4681347; doi:10.1111/1755-0998.12369)
Supplement: Supplementary file 6 — Appendix S2 An introduction to LDna: advanced. Tutorial which explains some of the more advanced features of LDna including suggestions on how to find appropriate values for the parameters φ and |E|min. A continually updated version is available from: https://github.com/petrikemppainen/LDna [file men0015-1031-sd6.pdf]

An introduction LDna: Advanced

Petri kemppainen, October 7, 2014

Introduction

This vignette provides a tutorial that demonstrates the use of LDna on a RAD sequence landscape genomics data set from a Southeast Asian mosquito malaria vector, *Anopheles baimaii*. This species has a widespread distribution extending from Northeast India, through Myanmar and into Thailand. Prior polytene chromosome mapping studies suggests that one polymorphic inversion is found on each of the five chromosome arms (2L, 2R, 3L, 3R and the X-chromosome) in *A. baimaii* and that they constitute up to 43% of their respective chromosomes [1-3]. Intraspecific inversion polymorphism is expected to result in high LD between large sets of loci that are identified by LDna from population genomic data sets. The full RAD sequence data set consists of 3828 SNPs from 184 samples and 91 geographical sites but here we only use a random subset comprising 25% of these loci called `r2.baimaii_subs`. This tutorial assumes you have read and understood the `LDna_basics` tutorial, accessed through: `vignette("LDna_basics", package="LDna")`.

Linkage disequilibrium network analysis

Installation

See `LDna_basics`

Explore data

To access sample data:

```
data(LDna)
LDmat <- r2.baimaii_subs
```

With only a matrix of pairwise LD values from a population genomics data set it is possible to plot networks at different LD thresholds to get an idea of how much 'LD clustering' exists in the data. This is achieved by the `plotLDnetwork` function and as can be seen below, at LD threshold 0.8 not much clustering can be seen. However, at 0.5 and 0.3 several distinct clusters can be seen. Between 0.5 and 0.3 some merging of large cluster has occurred, and the idea behind LDna is to find the exact LD thresholds when this happens such that these clusters can be extracted just before this happens. There will always exist at least one large cluster in a data set, given a low enough LD threshold value, but most often there will be several, sometimes up to hundreds, depending on LD threshold and data set.

```
par(mfcol=c(1,3))
plotLDnetwork(LDmat=LDmat, option=1, threshold=0.8)
plotLDnetwork(LDmat=LDmat, option=1, threshold=0.5)
plotLDnetwork(LDmat=LDmat, option=1, threshold=0.3)
```

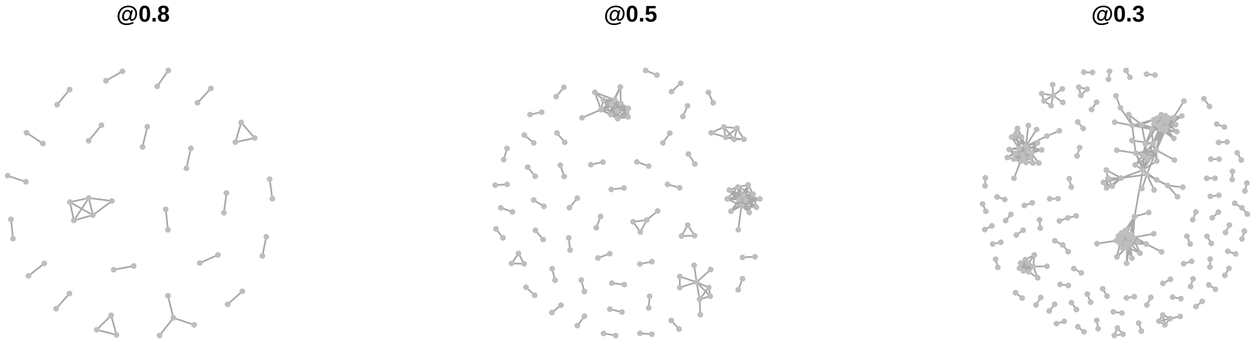

The LDna pipeline

The LDna pipeline is iterative involving several analysis steps. Once clusters have been identified they need to be visually explored by plotting networks as well as further analysed for instance by standard analyses of genetic structure and mapping to reference genome(s), if possible. Only then can the 'biological relevance' of an extracted cluster be assessed. In this tutorial we focus on a data set where we already know which clusters are associated with inversions from extensive downstream analyses. This tutorial will not cover the downstream analyses.

In practice, one would most likely explore the data set for a while through LDna, and once reasonably satisfied, proceed with simple downstream analyses. This information would then be fed back to a second round of LDna analyses, if necessary. This works well for reduced representation population genomic data sets with not too many loci (up to ~10000) such as RAD sequence data sets. If the signatures of LD are strong, not more than 1000 SNPs are sometimes necessary, as will be demonstrated here. If your data set is large, it is a good idea to start with a subsample (up to ~10000 SNPs and perhaps only from a subset of chromosomes, if this information is available) to get an overall idea about what factors are causing LD in the data before attempting to analyse the full data set, if necessary.

Produce raw data for LDna

The first step in LDna produces the raw data for all the subsequent analyses using the function `LDnaRaw`.

```
ldna <- LDnaRaw(LDmat)
```

Exploring cluster merger with decreasing LD threshold

Let's first have a look at the *single linkage clustering tree* (see `LDna_basics`), without being concerned about which clusters to extract. To do this we set the argument `extract` to `FALSE`, and the only additional arument we need to specify is $|E|_{min}$, controlled by `min.edges` (see `LDna_basics`). With `min.edges=1`, all clusters (with a minimum of two loci connected by one edge) are shown and it is relatively hard to see what is going on in the tree (see below). However, already with `min.edges=5`, four separate large branches can be seen. At `min.edges=18` only these four are visible; each of them representing clusters that remain large (above $|E|_{min}$) throughout a large range of LD thresholds.

```
par(mfcol=c(1,3))
extractClusters(ldna, min.edges = 1, plot.tree = TRUE, extract=FALSE)
extractClusters(ldna, min.edges = 5, plot.tree = TRUE, extract=FALSE)
extractClusters(ldna, min.edges = 18, plot.tree = TRUE, extract=FALSE)
```

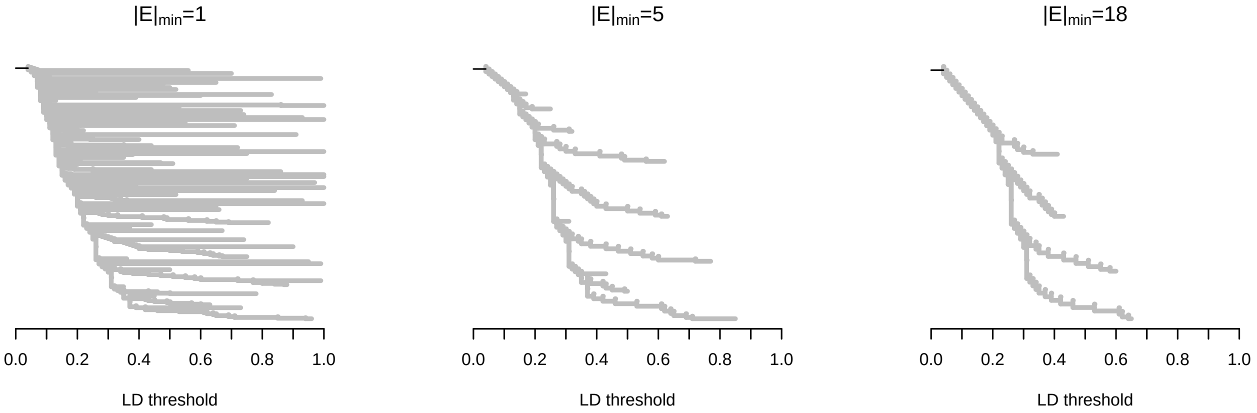

From the above trees we can predict that at LD threshold=0.4, four clusters with at least 18 edges, 6 clusters with at least five edges and well above ~50 clusters in total exist in the network. From the above we can also see that a 'significant' merger between two large clusters should happen at LD threshold ~ 0.3. If we plot networks again at LD thresholds 0.31 and 0.30 we can see that this is indeed the case.

```
par(mfcol=c(1,2))
plotLDnetwork(LDmat=LDmat, option=1, threshold=0.31)
plotLDnetwork(LDmat=LDmat, option=1, threshold=0.30)
```

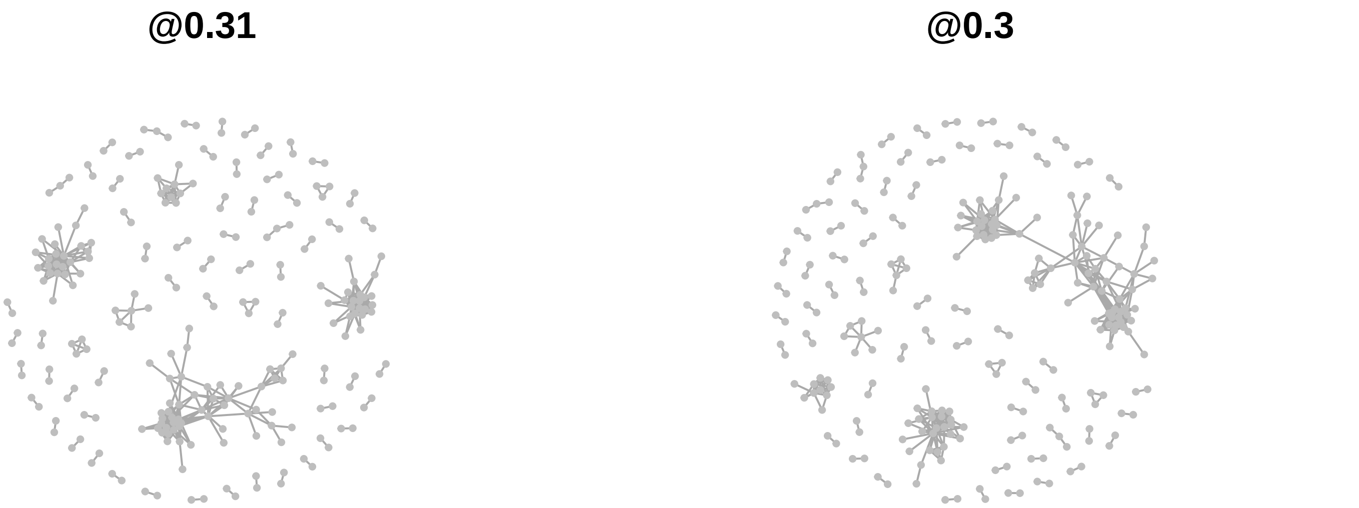

"Single outlier clusters" and "compound outlier clusters"

Outlier λ values imply the merging of large and distinct clusters comprising loci bearing different LD signals (see `LDna_basics`). However, as we can see from the above trees, there is a succession of cluster merging as the LD threshold is lowered. Along this succession only the first merger (moving form right to left along a branch in a tree) involves clusters were both are likely associated with separate distinct evolutionary factors or forces (see also `LDna_basics`). To distinguish that a no other *OCs* are nested within an *OC*, we call this *"single outlier cluster"* (*"SOC"*). When nested *OCs* occur within an *OC*, we denote it as a *"compound outlier cluster"* (*COC*) to indicate that it potentially contains loci bearing LD signals from more than two evolutionary factor or force. Whether this indeed is the case can, however, only be known from downstream analyeses. Normally we are only interested in *SOCs*, but for the purpose of illustration we will next demonstrate a tree where both *SOCs* and *COCs* are indicated. This is achieved by setting the argument `rm.COCs` to `FALSE` in the `extractClusters` function. In this figure blue colour indicates that the cluster in question is a *COC* rather

than a *SOC* (red). We set both `min.edges` and `phi` to relatively high values for this data set (18 and 4, respectively), such that we only get a few large *OCs* (this is covered in more detail below).

```
par(mfcol = c(1, 2))
clusters1 <- extractClusters(ldna, min.edges = 18, phi = 4, rm.COCs = FALSE,
    plot.tree = TRUE, plot.graph = FALSE)
```

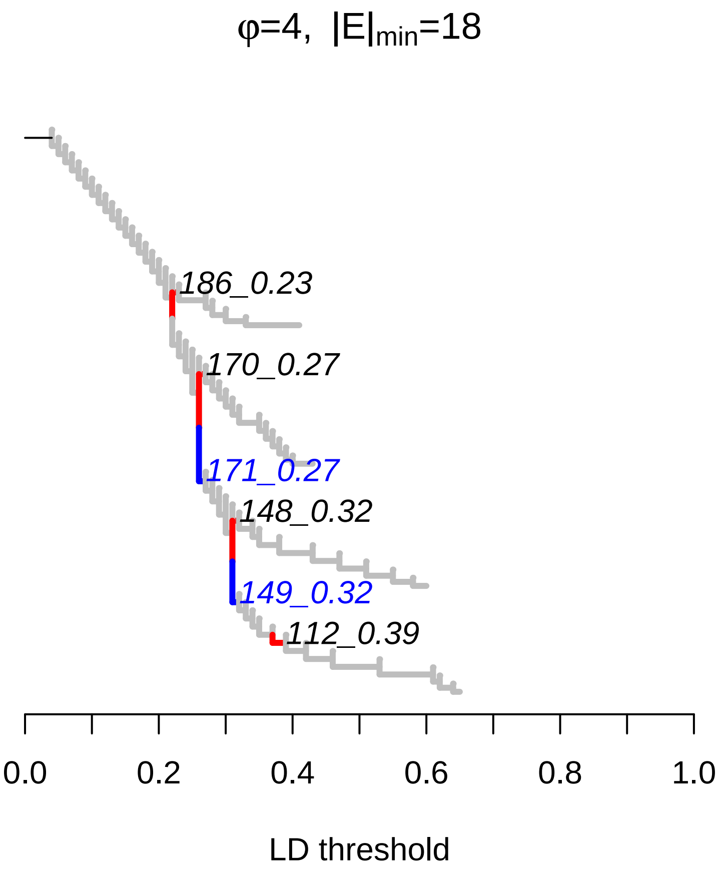

As can be seen from the above tree, *COC 149_0.32* includes loci from *SOC 112_0.39*, and *COC 171_0.27* in turn includes loci from *COC 149_0.32* and *SOC 148_0.32*. These results can further be summerised by the function `summaryLDna` (see `LDna_basics` for details), where the column: *"Type"* indicates whether an *OC* is a *COC* or a *SOC*.

```
summary1 <- summaryLDna(ldna, clusters1, LDmat)
summary1
```

```
          Type Merge.at nLoci  nE   lambda Median.LD MAD.LD
112_0.39   SOC     0.37    19  86 3.321917     0.386 0.1240
148_0.32   SOC     0.31    24 100 5.142672     0.269 0.1160
149_0.32   COC     0.31    51 174 3.751397     0.160 0.0700
170_0.27   SOC     0.26    31 135 5.139148     0.193 0.0763
171_0.27   COC     0.26   103 478 2.597112     0.075 0.0511
186_0.23   SOC     0.22    16  47 2.853319     0.195 0.0912
```

If we now plot all the extracted clusters (default settings), any *COC* will be colored blue and any SOC will be colored red. As can be expected, *SOCs* are generally more uniform in shape compared to *COCs*.

```
par(mfcol=c(2,3))
plotLDnetwork(ldna, LDmat, option=2, clusters=clusters1, summary=summary1)
```

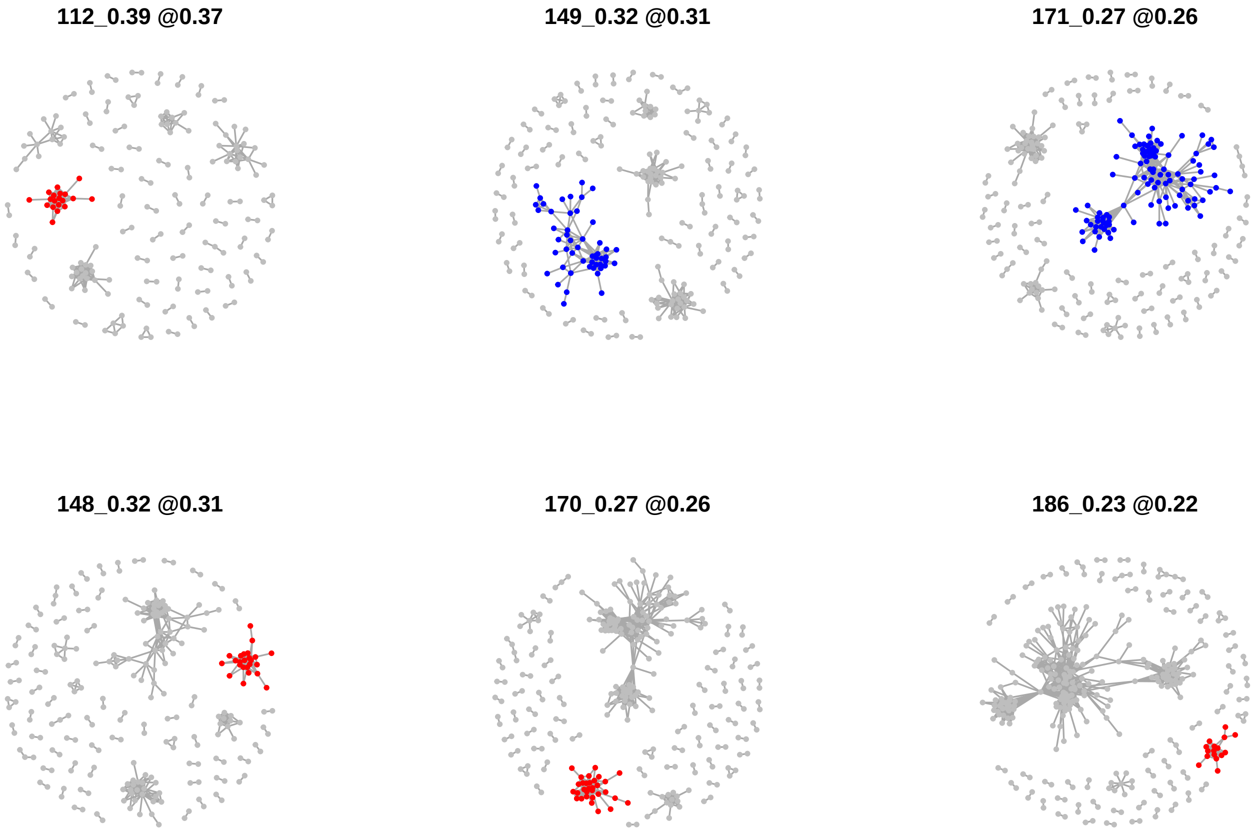

Identifying and extracting clusters, the effects of φ

Following from above analsyes `rm.COSs` is kept at `FALSE` (i.e both *SOCs* and *COCs* are considered), $|E|_{min}$ is kept at 18 but `phi` is changed from `4` to `2`. Thus, the limit for what is required for any cluster to become an *OC* is lowered. The effect of this is that two of the SOCs above (*148_0.32* and *SOC 112_0.39*) are now classified as COCs, as clusters *90_0.47* and *75_0.53* are also *OCs*. Below we show a graph where all λ values are ordered and any outlier values above λ_{lim} (see `LDna_basics`) are indicated. As can be seen, `phi`=2 gives a $\lambda_l im$=1.15 instead of 1.75 (λ for clusters *90_0.47* and *75_0.53* are 1.15 and 1.21, respectively).

```
par(mfcol = c(1, 2))
clusters_phi2 <- extractClusters(ldna, min.edges = 18, phi = 2, rm.COCs = FALSE,
    plot.tree = TRUE, plot.graph = TRUE)
```

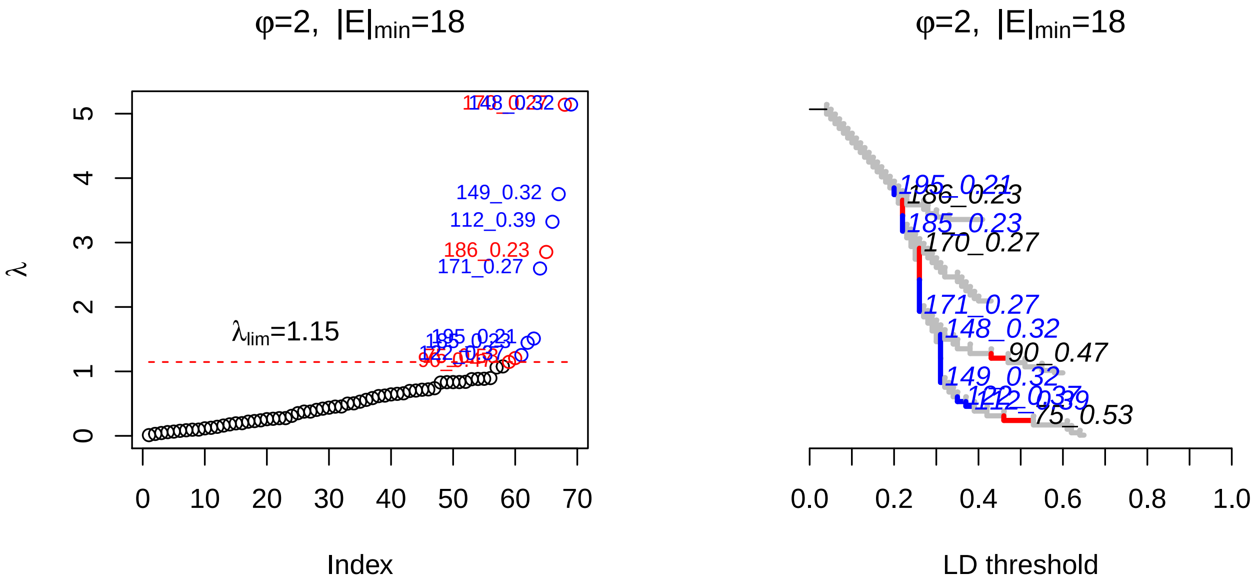

So which setting should we use for phi and which of the *OCs* (*148_0.32* or *90_0.47*) should we focus on? From the summary (below) we can see that the main differences between these two *OCs* are the size (with respect to numbers of loci), median LD and λ. Thus, with phi=2 we would identify a *SOC* (*OC 90_0.47*) from the branch in question that is smaller, have higher the median LD and lower λ relative to phi=4 (*OC 148_0.32*).

```
summary_phi2 <- summaryLDna(ldna, clusters_phi2[names(clusters_phi2) %in% c("90_0.47",
    "148_0.32")], LDmat)
summary_phi2
```

```
         Type Merge.at nLoci  nE   lambda Median.LD MAD.LD
90_0.47   SOC     0.43    14  53 1.147155     0.506  0.128
148_0.32  COC     0.31    24 100 5.142672     0.269  0.116
```

To further explore the differences between these two *OCs*, a few useful aspects of the plotLDnetwork function will be demonstrated. λ is a function of both the focal cluster (cluster before merger) and the cluster/loci it merges with (see the LDna_basics vignette), and we can use plotLDnetwork to specifically explore this. This is achieved by only showing the focal cluster and the cluster/loci it merges with by setting full.network to FALSE but also showing any clusters/loci the focal cluster merges with in the network by setting include.parent to TRUE. In addition, we can choose to show the clusters before or after merger via after.merger (TRUE for showing network after merger and FALSE for before, default is FALSE) as follows:

```
par(mfcol = c(1, 2))
plotLDnetwork(ldna, LDmat, option = 2, clusters = clusters_phi2[names(clusters_phi2) %in%
    c("90_0.47", "148_0.32")], summary = summary_phi2, full.network = FALSE,
    include.parent = TRUE, after.merger = FALSE)
```

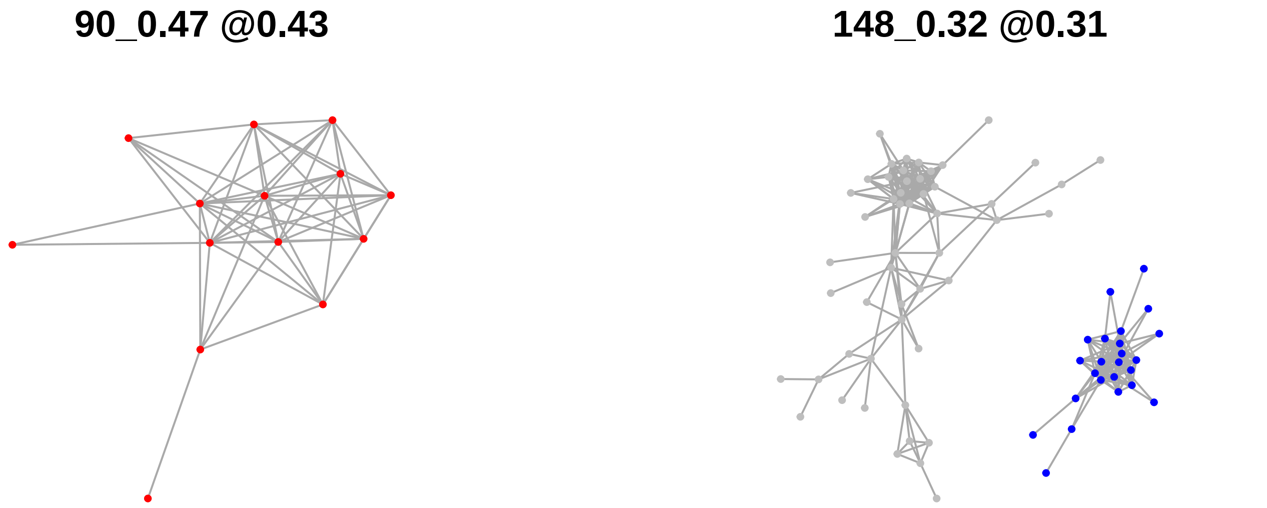

At first, the above seems strange as for *OC 90_0.47* only the focal cluster is shown (all vertices are colored red) in contrast to *OC 148_0.32* where both the focal cluster (blue) and the cluster it merges with (grey) are shown. When networks are plotted after merger (below) we can see that indeed *90_0.47* does not merge with any cluster(s) but with two separate loci. Although *90_0.47* only merges with two loci, there is a large difference in the pairwise LD values between them and the remaining loci, thus giving this cluster a relatively high λ-value. This is indicated by the large difference in LD threshold values when *OC 90_0.47* firstly appears (0.47) compared to when it merges with the two loci (0.43). Which one(s) of the two *OCs* do you then focus on? This question cannot fully be answered until some downstream analyses of both clusters have been performed. In this case we know that all loci in *OC 148_0.39* map to the same inverted region and thus `phi=4` makes the most sense if one is interested in extracting as many loci as possible.

```
par(mfcol = c(1, 2))
plotLDnetwork(ldna, LDmat, option = 2, clusters = clusters_phi2[names(clusters_phi2) %in%
    c("90_0.47", "148_0.32")], summary = summary_phi2, full.network = FALSE,
    include.parent = TRUE, after.merger = TRUE)
```

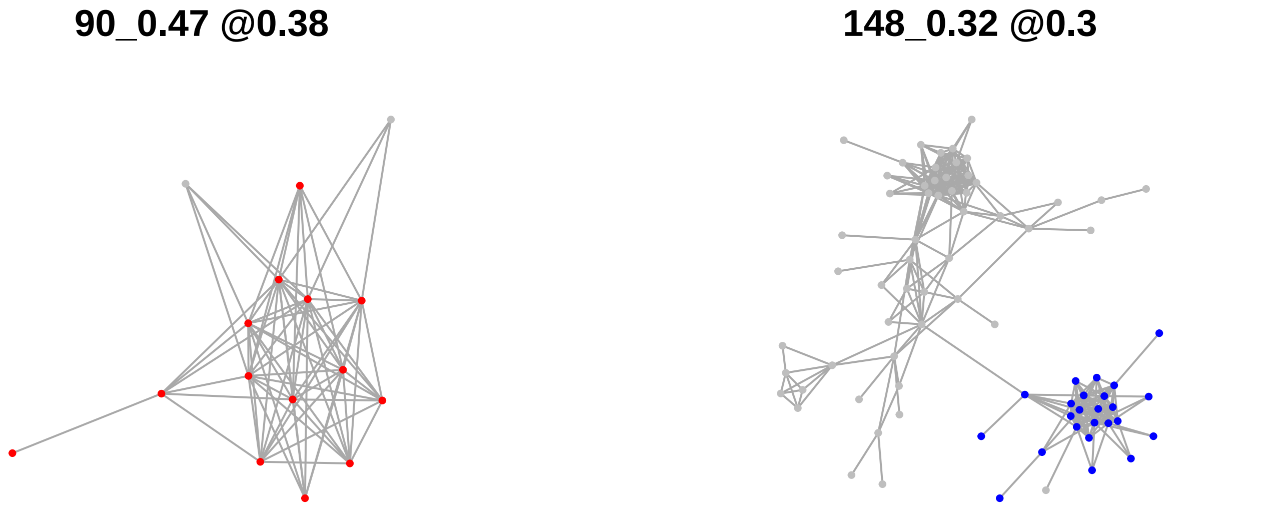

Based on our experience, we consider φ-values: 2-3 to be low, 3-5 to be medium and 5-8 to be high. As outlier λ values are relative to the median of all values in the tree this is likely to be true for any data set. Going below 2 and above 8 makes little sense except for the purpose of demonstration. In practice, for smaller data sets, the merging of 'biologically relevant clusters' may not cause exceptionally high λ-values (even relative to all other values in the data) and thus, in order to not miss such mergers, it makes sense to keep φ

relatively low for small data sets. With relatively low φ values you will always get at least one *SOC* from each 'large branch' in a tree. The risk with a 'too' low φ is that you may not be able to extract the maximum possible number of loci that are associated with a particular evolutionary factor/force. On the other hand, these loci will be in higher LD with each other and are thus more likely to be more closely associate with the evolutionary force/factor in question. If φ is set 'too' high there is a risk that a 'significant' merger will be missed leading to the identification of an *OC* that contains loci bearing LD signals from more than one distinct evolutionary factor/forces (i.e. it is in reality a *COC* not a *SOC*). However, downstream analysis can usually spot this.

Identifying and extracting clusters, the effects of $|E|_{min}$

In contrast to φ, what constitutes a low and a high value for $|E|_{min}$ is highly dependent on the number of loci in the data set, more specifically the resulting average SNP density in the genome. The more loci the more likely are SNPs to come from small blocks of non-recombining regions of the genome, so-called haplotype blocks. These are expected to result in many small but highly 'compact' clusters involving loci from very small genomic regions. However, generally LD breaks down rapidly as a function of physical/recombinatin distance in large outbreeding populations [4] and typical population genomics data set rarely reach such SNP densities. As a rough guideline, a good starting value for $|E|_{min}$ is 1-10% of the total number of SNPs in the data set. In practice, if the LD signals in the data are robust, a large span of different $|E|_{min}$ values will result in exactly the same *OCs*. Although small *OCs* may potentially also be important/interesting, $|E|_{min}$ is mainly a matter of convenience; you don't want to do all the downstream analyses for hundreds of *SOCs* (and the biggest ones are likely to be the most interesting). In the following example `min.edges` is deliberately set at a relatively low value (8).

```
clusters2 <- extractClusters(ldna, min.edges = 8, phi = 2, rm.COCs = TRUE, plot.tree = TRUE,
    plot.graph = FALSE)
```

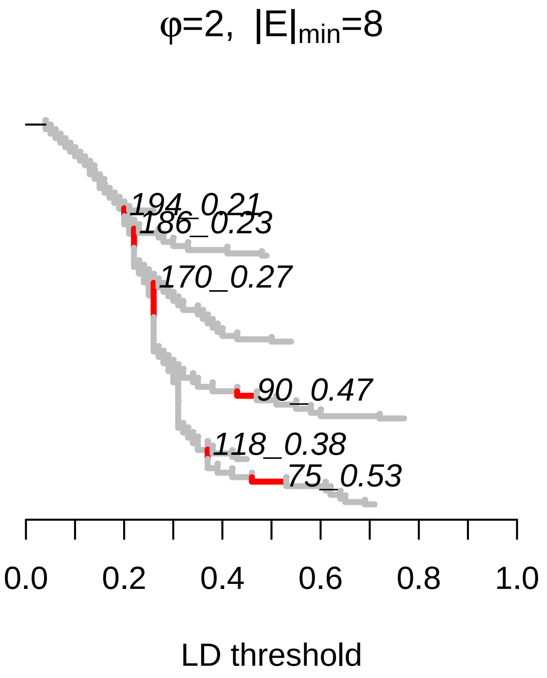

Here we focus on additional small clusters; *118_0.38* and *194_0.21* where we have not found any association with the five large inversions in this species. Downstream analyses of *OC 118_0.38* have revealed that this cluster is associated with overall population structure within this species (analyses not shown here). Because of the technique used to obtain this data (RADseq) some loci are likely to be physically close together, either because they are associates with the same restriction site or the SNPs come from the same RAD locus, and

this can also cause clusters in LD networks. Indeed, the 11 SNPs from *OC 194_0.21* only represent 7 unique RAD loci (first number before '.' indicates RAD locus) and mapping these loci to the reference genomes reveals that three of these pairs are associated with the same restriction cut-site. This demonstrates that LDna indeed is sensitive to finding even clusters in population genomic data sets that bear some biological relevance. However, it is important to pay attention to the details of the nature of the data when interpreting the results i.e. in the second example above, the cluster is likely an artifact of the nature of the data rather than a biologically interesting phenomenon.

```
clusters2$"194_0.21"
```

```
 [1] "L2425.1"  "L2425.4"  "L508.6"   "L7111.8"  "L5877.1"  "L1007.6"
 [7] "L1007.7"  "L3153.5"  "L4957.11" "L5758.9"  "L5758.18"
```

```
par(mfcol = c(1, 2))
summary2 <- summaryLDna(ldna, clusters = clusters2[names(clusters2) %in% c("118_0.38",
    "194_0.21")], LDmat)
plotLDnetwork(ldna, LDmat, option = 2, clusters = clusters2[names(clusters2) %in%
    c("118_0.38", "194_0.21")], summary = summary2)
```

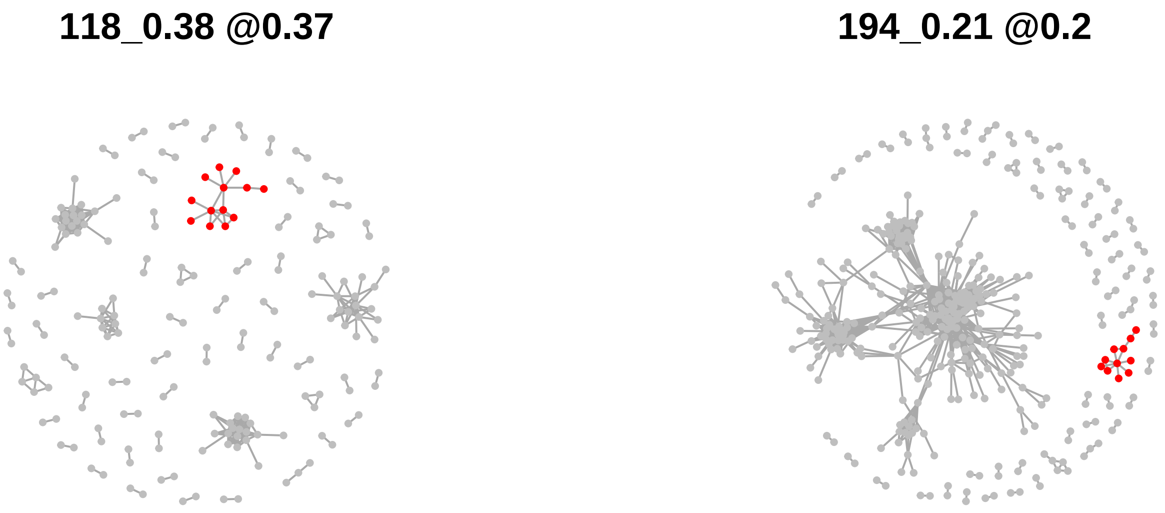

Final analysis

Having explored the data at sufficient depth we can run the full LDna pipeline with appropriate parameter values that result in the identification of only the 'most interesting' *OCs*, in this case those where downstream analyses have allowed us to reveal their association with inversions.

```
par(mfcol = c(1, 2))
clusters_final <- extractClusters(ldna, min.edges = 18, phi = 4)
```

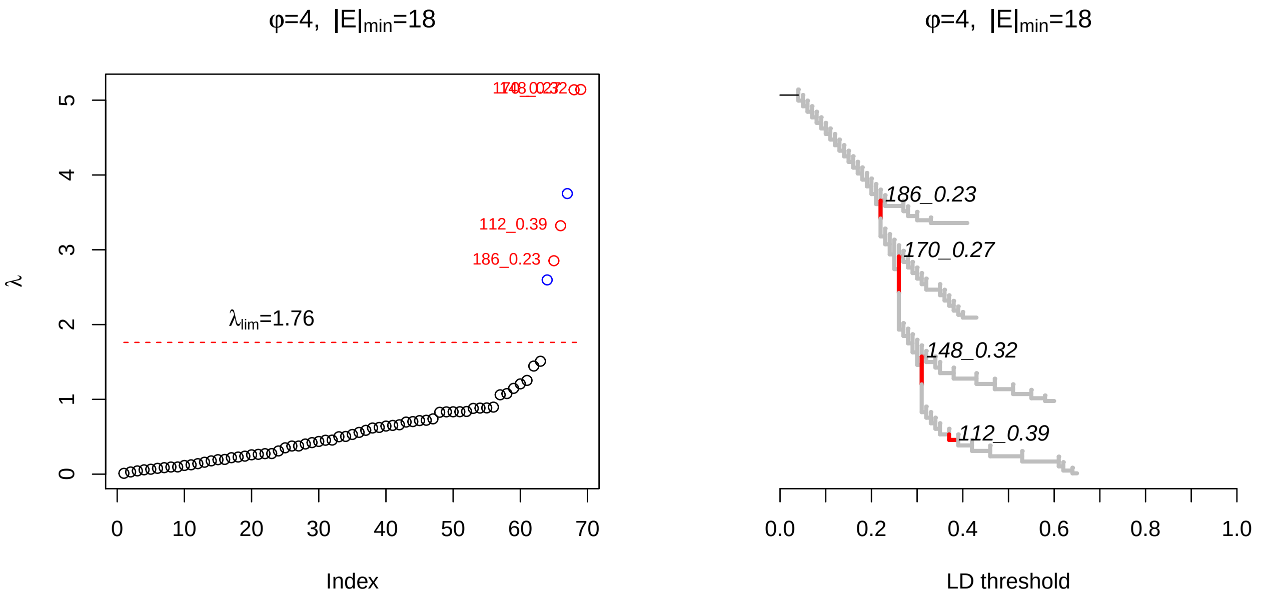


```
clusters_final
```

```
$`112_0.39`
 [1] "L4995.1"  "L9769.8"  "L3279.5"  "L665.6"   "L665.9"   "L1269.14"
 [7] "L289.1"   "L9364.4"  "L6075.5"  "L4731.7"  "L381.2"   "L6514.15"
[13] "L7312.9"  "L1191.7"  "L516.2"   "L9173.2"  "L567.1"   "L5083.16"
[19] "L7291.6"

$`148_0.32`
 [1] "L58.3"    "L3707.1"  "L3707.3"  "L6985.17" "L3489.23" "L2270.9"
 [7] "L4992.8"  "L625.9"   "L597.3"   "L1821.7"  "L3035.19" "L3941.13"
[13] "L3941.19" "L3924.1"  "L4845.15" "L634.4"   "L7339.6"  "L7339.9"
[19] "L4135.17" "L4885.10" "L4885.12" "L4807.7"  "L687.5"   "L9540.5"

$`170_0.27`
 [1] "L2458.13" "L4902.3"  "L2168.16" "L6555.6"  "L1447.2"  "L4313.16"
 [7] "L3779.7"  "L6759.1"  "L6759.2"  "L8312.4"  "L6045.3"  "L6581.17"
[13] "L5960.16" "L5631.22" "L2005.7"  "L4408.5"  "L4408.9"  "L5912.14"
[19] "L4387.1"  "L4387.7"  "L6235.3"  "L7327.4"  "L3680.16" "L5915.2"
[25] "L5957.5"  "L5710.3"  "L5710.9"  "L5710.17" "L3575.4"  "L827.3"
[31] "L4052.6"

$`186_0.23`
 [1] "L12.5"    "L6044.12" "L278.6"   "L229.1"   "L6834.11" "L372.10"
 [7] "L7024.9"  "L2434.11" "L6637.2"  "L6561.15" "L2530.9"  "L4821.4"
[13] "L8682.10" "L6554.6"  "L3493.6"  "L5268.4"
```

```
summary_final <- summaryLDna(ldna, clusters_final, LDmat)
summary_final
```

```
         Type Merge.at nLoci  nE   lambda Median.LD MAD.LD
112_0.39  SOC     0.37    19  86 3.321917     0.386 0.1240
148_0.32  SOC     0.31    24 100 5.142672     0.269 0.1160
```

```
170_0.27   SOC      0.26     31  135 5.139148      0.193 0.0763
186_0.23   SOC      0.22     16   47 2.853319      0.195 0.0912
```

```
par(mfcol=c(2,2))
plotLDnetwork(ldna, LDmat, option=2, clusters=clusters_final, summary=summary_final)
```

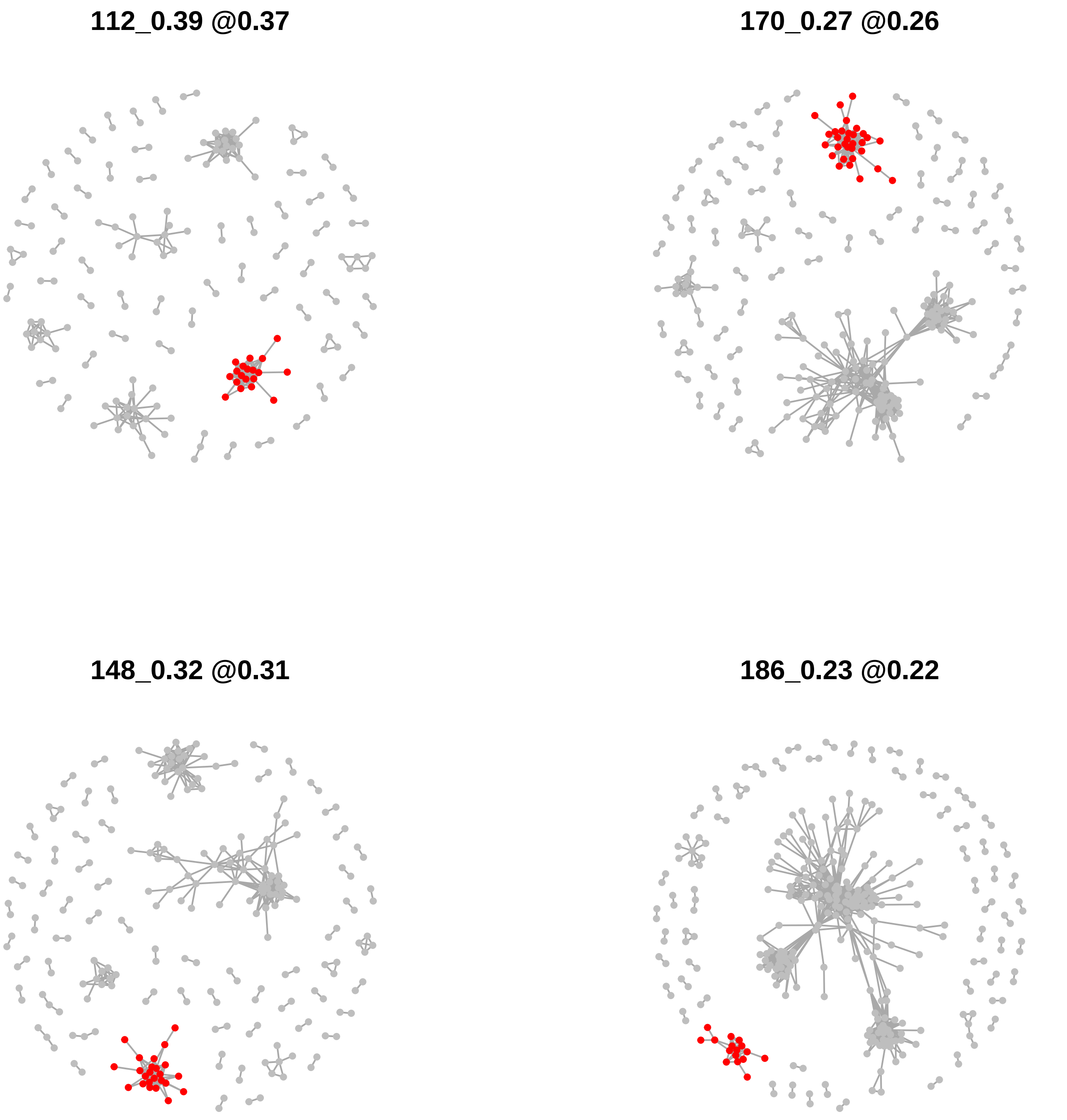

Concluding remarks on the choice of $|E|_{min}$ and φ

Although what constitutes a 'biologically relevant *SOC*' cannot be known *a priori*, there are three main reasons why such theoretical *SOCs* would not be identified from a given analysis: 1) $|E|_{min}$ is set too high such that the *SOC* in question falls below this value, 2) φ is set too low such that the *SOC* in question

is masked as a COC by allowing another SOC to be extracted at a higher LD threshold along the same 'branch', or 3) φ is set too high such that the λ value of the SOC in question is lower than the resulting λ_{lim}.

We recommend two alternative approaches for choosing which *OCs* to keep for downstream analyses. First, you can experiment with different values of $|E|_{min}$ and φ to find a setting that identifies exactly the *SOCs* you are interested in, perhaps after several rounds of downstream analyses of different *OCs*. Second, you can settle with a low value for both $|E|_{min}$ and φ and then 'cherry-pick' the *COCs* and *SOCs* (keeping `rm.COC=FALSE`) you are mostly interested for the downstream analyses. Or even analyse them all and then report which ones are the most interesting.

Note that exactly what cluster you in the end decide to extract for downstream analyses does not matter and you may not even be able to find one specific setting that allows you to extract all the *OCs* you are interested in. What matter is what the downstream analyses show; there is nothing wrong with initially including many different *SOCs* and *COCs* in the downstream analyses and only then decide which of them make the most sense, in fact this is highly recommended. The most intersting mergers will already be obvious from the single linkage clustering tree, and **LDna** is only a way to access these in a convenent way without having to analyse all different clusters in the data.

References

1. Baimai V, Poopittayasataporn A, Kijchalao U (1988) Cytological differences and chromosomal rearrangements in four members of the Anopheles dirus complex (Diptera: Culicidae). Genome 30: 372–379.
2. Baimai V, Thu MM, Paing M (1988) Distribution and chromosomal polymorphism of the malaria vector *Anopheles dirus* species D. The Southeast Asian J Trop Med Public Health 19: 661–665.
3. Poopittayasataporn A, Baimai V (1995) Polytene chromosome relationships of five species of the Anopheles dirus complex in Thailand. Genome 38: 426–434.
4. Slate J, Pemberton JM (2007) Admixture and patterns of linkage disequilibrium in a free-living vertebrate population. J Evol Biol 20: 1415–1427.
