# Supplementary material for: Linkage disequilibrium network analysis (LDna) gives a global view of chromosomal inversions, local adaptation and geographic structure
Source: Mol Ecol Resour. 2015 Jan 21;15(5):1031–45. doi: 10.1111/1755-0998.12369 (PMC4681347; doi:10.1111/1755-0998.12369)
Supplement: Supplementary file 7 — Appendix S3 Linkage disequilibrium network analysis (LDna) on simulated data [file men0015-1031-sd7.pdf]

Appendix S3 Linkage disequilibrium network analysis (LDna) on simulated data

Methods

Here we demonstrate two basic aspects of linkage disequilibrium network analysis (LDna) using simulated data (summarized in Fig. 1). The first approach, ①, aims to demonstrate what can be expected from linkage disequilibrium (LD) networks when admixture LD is artificially created by pooling individuals from different populations (with no gene flow) prior to calculating LD. The second approach, ②, aims to demonstrate how gene flow between populations affects clustering in LD networks. An exhausting simulation experiment for testing LDna is outside the scope of this study, and this appendix is only intended as a demonstration of how a common source of LD (population structure; with and without gene flow) causes clustering in LD networks. All simulated data sets were generated with the software 'fastsimcoal2' (Excoffier & Foll 2011; Excoffier *et al.* 2013).

All simulations involved three chromosomes of 1Mb each. Recombination rate between adjacent loci within each chromosome was set to 6.9 x 10^{-07}, corresponding to a 50% probability of one or more recombination events per chromosome. The mutation rate was set to 1 x 10^{-07} with no transition bias. We sampled 50 haploid chromosomes from each population (25 diploid individuals) and 3000 loci for each data set in approach ① and 1000 loci for each data set from approach ② (approach ① required more loci than ② in order to detect all relevant clusters). All loci were bi-allelic and had a minimum allele frequency >10%. Effective population sizes (N_e) and divergence times and migration rates (expressed as effective number of migrant gene copies per locus and generation, N_e*m, where m is the probability of a gene copy coming from another population each generation) are given in Fig. 1. LD was calculated as for all other data sets in this study (see main text for details). Input files for the simulations, simulated data sets, the corresponding pairwise r^2 matrices and R-code for the analyses are available from: http://doi.org/10.5061/dryad.2t764.

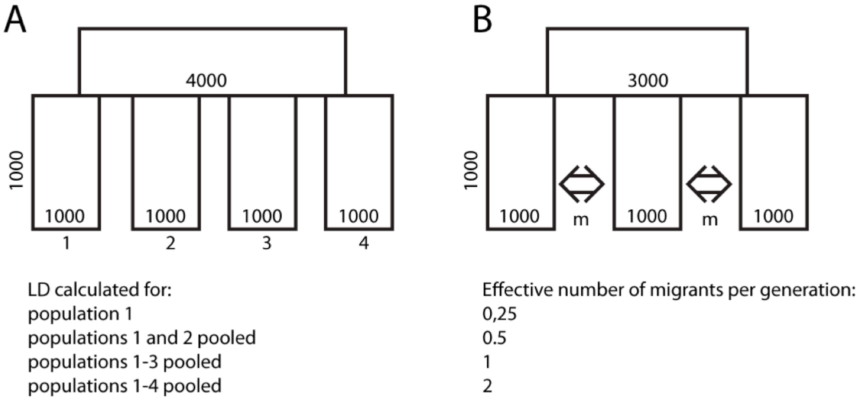

Fig. 1. Two simulation approaches were used to demonstrate clustering in LD networks. First (A), an instantaneous split was simulated after which artificial LD was created by pooling individuals from 1-4 populations prior to estimating LD (approach ①). Second (B), an instantaneous split into three populations was simulated, where N_e*m varied between 0.25 and 2 (approach ②). Additional details of the simulations are given in the figure and in the main text of this Appendix.

Results and Discussion

Artificial admixture LD

As seen from the results of simulation approach ① (Fig. 2), when LD is calculated for a single panmictic population, clusters with more than 20 edges start to form at LD threshold ~≤ 0.5 but no particular cluster is obviously larger than any other. This LD is caused by stochastic factors (i.e. the Hill-Robertson effect; Hill & Robertson 2007) and the founder event following the reduction of Ne from 4000 to 1000 at the time of population divergence (see Fig. 1). No clear SOCs are expected from this data set (note that SOCs can always be found by setting the parameter values for λ and φ sufficiently low).

With two populations pooled prior to calculating LD, the resulting artificial admixture LD causes one distinct cluster in the data set that continually grows with decreasing LD threshold. As the detection of SOCs requires the merging of at least two large and distinct clusters (relative to all merging events in the clustering tree, see main text for details), no clear SOCs can be expected from LDna from this data set (but see note above).

With artificial admixture LD from three populations, three separate and clear clusters can be discerned (Fig. 2). These are caused by drift fixing different alleles in different combinations in the populations that subsequently causes distinct patterns of LD in the data (this generates three easily identifiable SOCs as demonstrated in Fig. 2, main text). For instance, all loci where a single derived alleles become fixed in one population but remains ancestral in the other two, will be in LD with each other and cause a distinct cluster (See Fig. 2, main text).

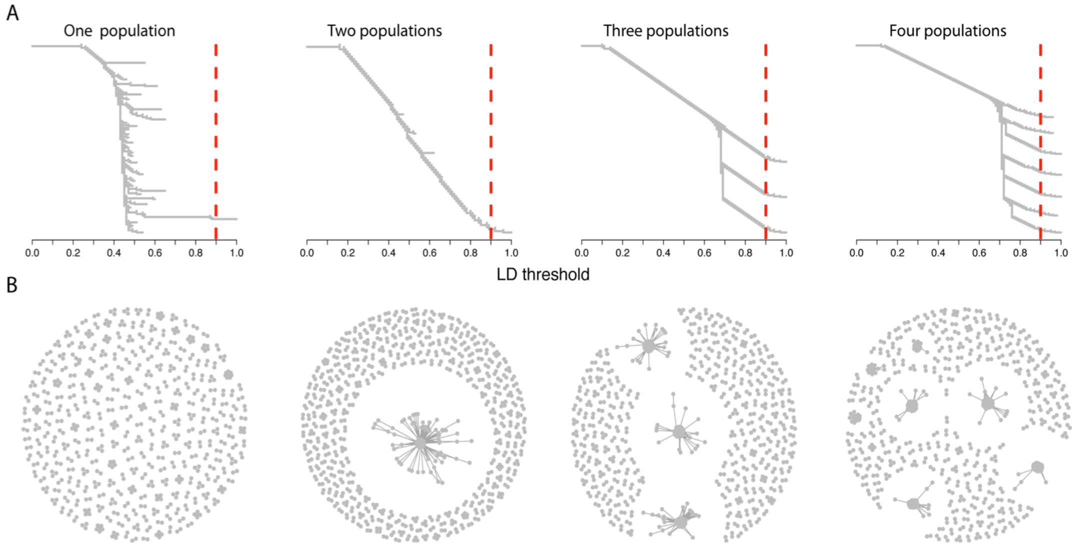

Fig. 2. LDna and artificial admixture LD - simulation approach ①. An instantaneous split into four populations was simulated and LD was calculated when individuals from 1-4 of these populations were pooled prior to calculating LD (see Fig. 1 and main text of this Appendix for details). (A) Shows clustering trees ($|E|_{min}$=20) for these four data sets and (B) shows LD networks at LD threshold=0.9 as indicated by the red lines in (A).

With artificial admixture LD cased by four populations, there are four ways in which one population may be separated from the other three by a single derived allele (in the same way as there are 3 ways for three populations in Fig. 2); this results in the four large clusters in the right-hand panel of Fig. 2B. However, there are also three ways in which two populations may be separated from the other two by a shared derived allele; this results in the three smaller clusters in the right-hand panel of Fig. 2B. This will result in seven easily identifiable SOCs in the data (e.g. at parameter settings: φ=5 and λ=20, not shown). More generally, the number of LD clusters that potentially generate SOCs in the data is given by $2^{(n-1)}-1$ where n is the number of populations.

Population structure with gene flow

In approach ② an instantaneous split into three populations was simulated followed by different levels of migration rates (N_e*m). Figure 3 shows that with increasing rates of recombination between chromosomes of different ancestry (gong left to right in the figure), LD clusters caused by population structure becomes less distinct. Nevertheless, even with N_e*m=1 three different clusters can clearly be discerned, and at N_e*m=2 some clustering in the LD networks is still present. In all cases, the clusters visible in Fig. 3 result in SOCs with the parameter setting: φ=5 and λ=20, except in the data set with N_e*m=2, where only two SOCs are identified by LDna at this setting (data not shown). In a principal component analysis all loci from SOCs separate individuals based on the population they originate from (data not shown).

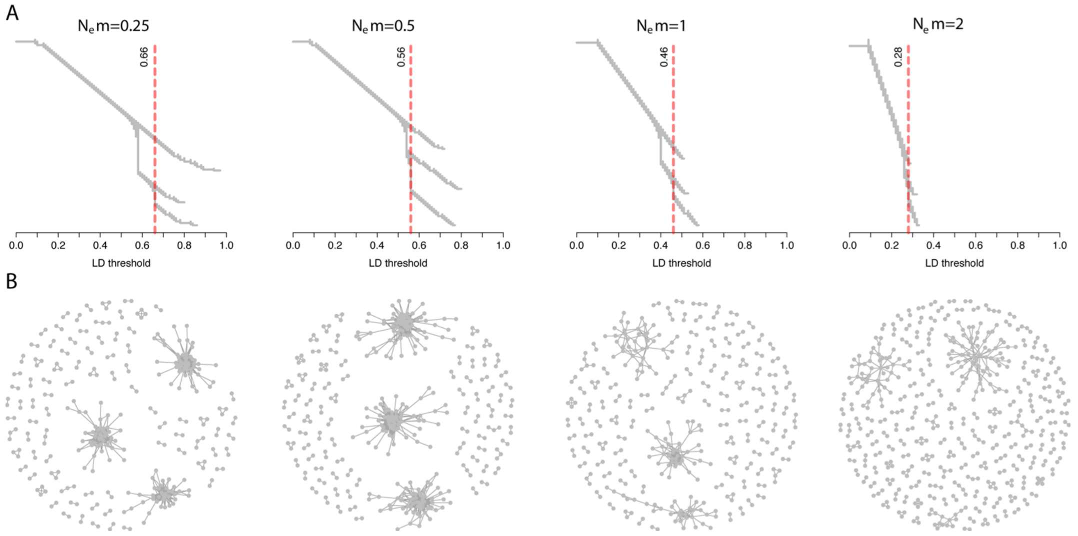

Fig. 3. Recombination breaks down LD generated by population structure - simulation approach ②. An instantaneous split into three populations was simulated with N_e*m varying between 2 and 0.25 (see Fig. 1 and main text of this Appendix for details) as indicated above figures. (A) Shows clustering trees with $|E|_{min}$=20. (B) Networks are shown at an LD threshold just before any large clusters merge as indicated by red lines in (A). Note that the LD thresholds are not the same for the four different networks.

References

Excoffier L, Foll M (2011) fastsimcoal: a continuous-time coalescent simulator of genomic diversity under arbitrarily complex evolutionary scenarios. *Bioinformatics (Oxford, England)*, **27**, 1332–1334.

Excoffier L, Dupanloup I, Huerta-Sánchez E, Sousa VC, Foll M (2013) Robust demographic inference from genomic and SNP data. *PLoS Genetics*, **9**, e1003905.

Hill WG, Robertson A (2007) The effect of linkage on limits to artificial selection. *Genetical research*, **89**, 311–336.
