# Supplementary material for: Linkage disequilibrium network analysis (LDna) gives a global view of chromosomal inversions, local adaptation and geographic structure
Source: Mol Ecol Resour. 2015 Jan 21;15(5):1031–45. doi: 10.1111/1755-0998.12369 (PMC4681347; doi:10.1111/1755-0998.12369)
Supplement: Supplementary file 8 — Appendix S4 Anopheles baimaii RAD sequences data set and three-spined sticklebacks SNPs data set preparation [file men0015-1031-sd8.pdf]

Appendix S4 Data-set preparation of *A. baimaii* and three spined stickleback data-sets

Anopheles baimaii landscape genomic sample collection and DNA extraction
Individual mosquitoes were collected between 2005-2011 from Myanmar and northeast India by overnight light traps (Sarma *et al.* 2012) and preserved in silica gel until DNA extraction. DNA was extracted using a modified TENS/phenol/chloroform DNA extraction protocol (Surendran *et al.* 2013). DNA yield and quality was assessed with Quant-iT Pico Green double stranded DNA Assay kit (Invitrogen) on an OPTIMA plate reader (BMG LABTECH) and by running the samples on 1.5% agarose gels. A total of 224 individual DNA samples were chosen for the landscape genetics RAD libraries based on their location and DNA quality.

Anopheles baimaii RAD sequence library preparation
RAD sequence libraries were prepared following the protocol of Baird et al. ((Baird *et al.* 2008) using an *SbfI* high fidelity restriction enzyme (New England Biolabs), with some modifications to account for the low quality and yield of DNA from the field collected samples in the landscape genomics data-set, as follows. 50-100 ng of DNA per sample was used for the restriction digestion and P1 adapter ligation was performed in 10-12 µl reaction volumes (in 96 well 200 µl PCR-plates) using 1 µl of 0.5 µM adapter. Sixty-four individually barcoded P1 adapters were used with 5 bp barcodes that differed by at least two bases. After P1-ligation, all samples for a library were pooled, and shearing was conducted with a Covaris S2 in 6x16mm Micro Tubes (COVARIS) and sonication. The DNA pools were subdivided into 5 and different shearing regimes were used for different subsets: two subsets used 15 s shearing, one 30 s and two 45s (duty cycle = 5%, intensity=3, 200 cycles/burst). From each of these we selected DNA fragments of 300-650 bp. This approach was intended to obtain an even representation of all samples in the final library since the original sample DNA was sheared to varying degrees. In every purification step a variable quantity of DNA is lost thereby increasing the variation of DNA concentration among individuals in the final RAD library, and this problem is expected to increase with the number of samples in the pool. To help average out these changes throughout the protocol, all purification steps were split into five replicates and the eluent was re-pooled after each step. Final selective amplification for each library was conducted in 40 replicate PCR reactions of 15 µl each for 18 cycles, using FUSION High-Fidelity Taq (FINNZYMES) to reduce the occurrence of PCR duplicates. In total five libraries were sequenced on an Illumina HiSeq 2000 platform (2 × 100 bp paired-end sequencing) at GenePool (University of Edingburgh).

Anopheles baimaii quality filtering and SNP calling
The libraries were de-multiplexed using the process_radtags script from the Stacks pipeline version 0.99991 (Catchen *et al.* 2011). This checks that both the restriction cut site and barcode are intact and, if possible, corrects errors. Only reads with a raw phred score of 10 within a sliding window of 15% of the total read length were kept, thus allowing for some errors in the retained sequences. PCR duplicates were identified by almost identical (to allow for some sequencing

error) paired-end reads and removed by the script purge_PCR_duplicates.pl available from: https://github.com/claudiuskerth/scripts_for_RAD/blob/master/purge_PCR_duplicates.pl. Denovo_map.pl was used to execute the stacks pipeline. For the landscape genomics data-set we entered all individuals as one population (i.e. all individuals were used to create a catalog of loci, see Stacks documentation for details). For the LD map data-set (Appendix S4, Supporting Information) we entered the P1 and F1 individuals as parents (only loci present in these individuals were entered into the catalog) and all F2 individuals as progeny whose genotypes were matched against the catalog loci allowing Stacks to correct progeny genotypes, where possible (see Stacks documentation for details). We enabled the removal or break up of over-merged loci (see Stacks documentation for details). We set a minimum stack depth of six reads per allele, allowed three mismatches between alleles to create a locus for an individual and three mismatches between loci between individuals to form the final catalog loci as these settings were found to produce a good balance between under and over-merged loci in our data-set. Data were exported using the export_sql.pl script where all loci with less then 150 (out of 221) genotypes were discarded. Subsequent filtering of the RAD data was done with custom R scripts.

The raw landscape genomics RAD data-set from *A. baimaii* comprised 3666 loci from 221 individuals with 20.6 % missing genotypes and this was filtered as follows. Any genotype with a stack depth below 18 was removed (60030 genotypes). If the read count for an allele was less than 5 % of the maximum read count for any allele for that genotype it was regarded as an error and the allele was removed from the genotype (153 corrections). Genotypes with more than two sequences at a particular locus indicate duplicated genes. If, after correction of errors, genotypes still included more than two alleles, the genotype was flagged as 'disploid' and removed (1951 genotypes). If more than 5 % of the genotypes for a locus were disploid the whole locus was removed (42 loci). In heterozygous individuals the read count for one allele was allowed to be four or fewer times more common than the other allele, otherwise it was flagged as 'unreliable' and removed (364 genotypes). If more than 5 % unreliable genotypes were found per locus (one locus), the whole locus was excluded. Finally, a maximum of 25 % of missing genotypes was allowed across any locus (658 loci removed) and a maximum of 50 % of missing genotypes was allowed across any individual (37 individuals removed).

Twenty-one genomes stickleback genome data
Single nucleotide polymorphism data from 21 stickleback genomes were downloaded from http://sticklebrowser.stanford.edu (Jones *et al.* 2012). Sample sizes were: Pacific freshwater: n=7; Atlantic freshwater: n=4; Pacific marine: n=5 and Atlantic marine: n=5. Only SNPs with a maximum of two missing genotypes were used. Initial analyses suggested that many loci came from repetitive regions as indicated by extremely high observed heterozygosities. This led to high estimates of LD between these loci causing a distinct cluster in LDna. To avoid this, any SNPs with observed heterozygosity higher than 0.5 were removed. Another effect of using such a low threshold for observed heterozygosity was to bias the data towards informative SNPs i.e. those having

high allele frequency differences between different populations, ecotypes or chromosomal rearrangements that result in high LD. In LDna the inclusion of a single individual divergent at many loci can be enough to cause a cluster. This was common in this Stickleback data-set as it comprised relatively few individuals sampled from a large geographical range; in PCA these clusters will separate one individual from all other samples. In this study we were only interested in general causes of LD and so removed all loci with a minor allele frequency below 20%.

Data-sets at various stages of quality filtering for both *A. baimaii* and the three-spined stickleback are available from Dryad:
http://doi.org/10.5061/dryad.2t764

References:

Baird NA, Etter PD, Atwood TS *et al.* (2008) Rapid SNP Discovery and Genetic Mapping Using Sequenced RAD Markers. *PLoS One*, **3**, e3376.

Catchen JM, Amores A, Hohenlohe P *et al.* (2011) Stacks: Building and Genotyping Loci De Novo From Short-Read Sequences. *G3 (Bethesda, Md.)*, **1**, 171–182.

Jones FC, Grabherr MG, Chan YF *et al.* (2012) The genomic basis of adaptive evolution in threespine sticklebacks. *Nature*, **484**, 55–61.

Sarma DK, Prakash A, O'Loughlin SM *et al.* (2012) Genetic population structure of the malaria vector Anopheles baimaii in north-east India using mitochondrial DNA. *Malaria journal*, **11**, 76.

Surendran SN, Sarma DK, Jude PJ *et al.* (2013) Molecular characterization and identification of members of the Anopheles subpictus complex in Sri Lanka. *Malaria journal*, **12**, 304.
