# Supplementary material for: Linkage disequilibrium network analysis (LDna) gives a global view of chromosomal inversions, local adaptation and geographic structure
Source: Mol Ecol Resour. 2015 Jan 21;15(5):1031–45. doi: 10.1111/1755-0998.12369 (PMC4681347; doi:10.1111/1755-0998.12369)
Supplement: Supplementary file 9 — Appendix S5 Anopheles baimaii linkage map preparation [file men0015-1031-sd9.pdf]

Appendix S5 *Anopheles baimaii* linkage map preparation

Linkage map cross

Live *A. baimaii* larvae were collected from sample sites in Mudon and Dawei (Fig. S1, Supporting Information), south Myanmar in July 2011, reared to adulthood in the laboratory and crossed for one generation within each site. Twelve randomly chosen adult individuals from these crosses (one parent from each population) were selected as P1 parents for the linkage map crosses. For each cross 12 F1 individuals were intercrossed to produce the F2 generation, each of these producing ~100 offspring. One of these crosses was chosen for the RAD linkage map from which four separate F1*F1 families were used with 18-19 F2 individuals each. DNA was extracted as for the landscape genomics samples above.

RAD sequence preparation and quality filtering

Restriction-site associated DNA sequence data-set for the linkage map cross was generated as described in Appendix S4 (Supporting Information). The raw RAD data for the linkage map (96 individuals and 2938 RAD loci) were initially filtered as the landscape genomics data-set (Appendix S4, Supporting Information, with similar values for removed genotypes/alleles/loci). In addition, to find linkage map informative loci only those complying with the following criteria were kept: 1) at least one genotype from the P1 parents was present, 2) all genotypes in the F1 individuals were heterozygous, 3) by comparing the F1 genotypes with the P1 genotypes we could predict which alleles came from which parent and 4) based on this, the parents were fixed for different alleles and could therefore be used to construct linkage maps. Lastly, any loci that significantly deviated from the expected segregation ratio of 1:2:1 in the F1 individuals (at α=0.05, as tested by χ^2-tests) for the genotypes: aa:ab:bb (where a alleles were unique for one parent and b alleles were unique for the other parent) were also excluded. Loci for building a linkage map for the X chromosome were identified as for the autosomes except here we used the expectation that loci were heterozygous in the female F1 individuals and homozygous in the male F1 individuals. Finally, any individuals with more than 25% missing genotypes were removed resulting in data-sets comprising 318 linkage map informative loci from 51 F1 individuals for the autosomes and 88 loci from 47 F1 individuals for the X-chromosome.

Linkage map construction

The filtered loci were assigned to linkage groups by the *independence LOD score* in Joinmap, version 4.1 (Van Ooijen 2011) using default parameters. This was done separately for the autosomal loci and the X linked loci and only markers associated with each other at LOD score above 10 were assumed to belong to the same linkage group. Linkage order for each linkage group was calculated using the *maximum likelihood mapping algorithm* using default parameters. Any loci with nearest neighbor stress parameter above two were removed (two loci) as high values indicate that a locus is at the wrong position. The resulting linkage map was further analyzed in the R-package '*R/qtl*' (Broman *et al.* 2003) where we obtained the maximum likelihood estimate of the error rate (see *R/qtl* documentation for details), and used this error rate to calculate the final map

with the *Lander-Green algorithm* and *Haldane* mapping function. Recombination fractions and LOD scores were calculated for each pair of loci and plotted in the order they appeared in the linkage map to visually inspect the order of loci for any obvious errors.

Loci could be assigned to two linkage groups of 194 loci (linkage group I) and 128 loci (linkage group II), respectively, for the autosomes and one linkage group of 88 loci for the X-chromosome (Fig. 1 and Fig. 5, main text). From this we cannot assign these autosomal linkage group to the previously named chromosomes 2 and 3. Linkage map data-sets are available from Dryad: http://doi.org/10.5061/dryad.2t764

References:

Broman KW, Wu H, Sen S, Churchill GA (2003) R/qtl: QTL mapping in experimental crosses. *Bioinformatics (Oxford, England)*, **19**, 889–890.

Van Ooijen JW (2011) Multipoint maximum likelihood mapping in a full-sib family of an outbreeding species. *Genetics research*, **93**, 343–349.

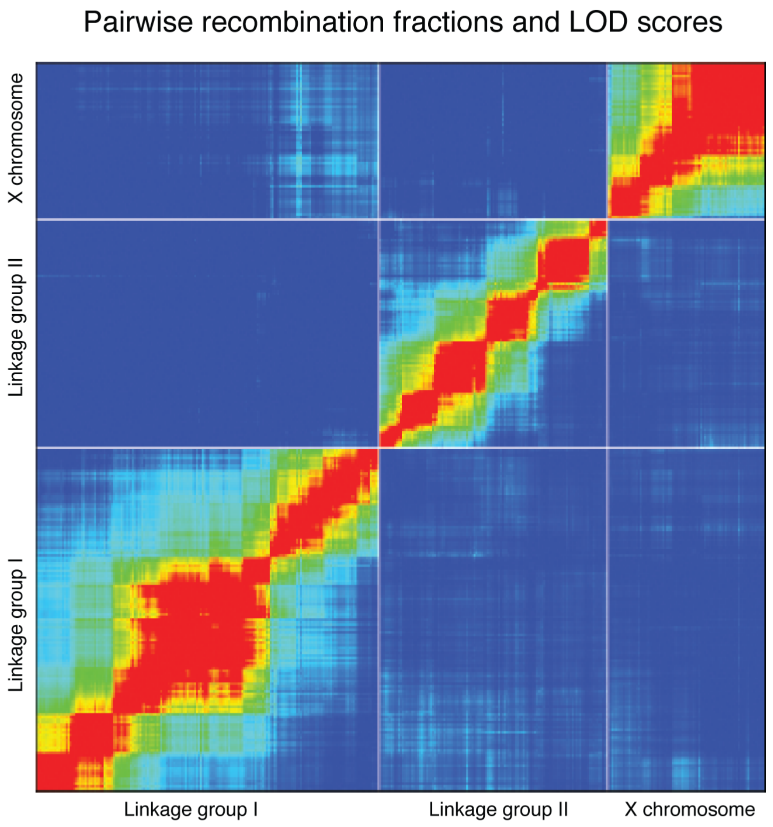

Fig. 1 Recombination fractions and LOD scores for *A. baimaii* linkage maps. Upper diagonal shows recombination fractions and lower diagonal shows LOD scores for the *A. baimaii* linkage map.
